# Implementation and effectiveness of intermittent preventive treatment in school aged children using dihydroartemisinin-piperaquine to reduce malaria burden: an implementation research of a cluster randomised trial in Tanzania

**DOI:** 10.1016/j.eclinm.2025.103628

**Published:** 2025-11-07

**Authors:** Geofrey Makenga, Bruno Mmbando, Misago D. Seth, Vito Baraka, Daniel P. Challe, Filbert Francis, Athanas D. Mhina, Edwin Liheluka, Daniel T.R. Minja, Mercy Chiduo, George Mtove, Celine Mandara, Samwel Gesase, Method D. Segeja, Mathias Kamugisha, Paul M. Hayuma, Joyce R. Mbwana, Hillary Sebukoto, Anangisye Malabeja, Juma B. Tupa, Sarah J. Ngede, Abdallah Lusasi, Frank Chacky, Anna David, Sumaiyya G. Thawer, Ally Mohamed, Sijenunu Aaron, Samwel Lazaro, Fabrizio Molteni, Alex Nkayamba, Hilde Bastiaens, Jean-Pierre Van geertruyden, John P.A. Lusingu

**Affiliations:** a National Institute for Medical Research, Tanga Centre, Tanga, Tanzania; b National Malaria Control Programme (NMCP), Tanzania; c Swiss Tropical and Public Health Institute, Allschwill, Switzerland; d University of Basel, Basel, Switzerland; e Tanzania Medicine and Medical Devices Authority (TMDA), Tanzania; f Global Health Institute, University of Antwerp, Antwerp, Belgium; g Centre for Translational Medicine and Parasitology, Institute of Medical Microbiology and Immunology, University of Copenhagen, Denmark

**Keywords:** Effectiveness, Dihydroartemisinin-piperaquine, Implementation research, Cluster randomised controlled trial, Feasibility, Malaria chemoprevention, IPTsc

## Abstract

**Background:**

In malaria endemic areas, malaria is a major contributor for half of the mortality in school-aged children (5–15 years old). Most infections are asymptomatic, contributing to anaemia, poor cognitive development, and reduced school performance. School-aged children often show low adherence to preventive and curative measures, and malaria control programs rarely target this group. We evaluated the pilot implementation of intermittent preventive treatment of malaria in schoolchildren (IPTsc) using Dihydroartemisinin-Piperaquine (DP) delivered by schoolteachers, to assess the implementation feasibility and effectiveness on asymptomatic and clinical malaria in a high endemic area in Tanzania.

**Methods:**

This study used a cluster-randomised design in which each ward (cluster) was assigned to implement IPTsc or serve as a control. All schoolchildren in primary schools under the intervention arm received three annual rounds of DP aligned with malaria transmission seasons. The primary implementation end point was IPTsc coverage, defined as the proportion of enrolled schoolchildren completing the full DP therapeutic course. For the effectiveness, 12 wards were randomly selected per arm, and one school per ward contributed outcome data. The primary effectiveness endpoints were (1) protective effectiveness of IPTsc on clinical malaria incidence and (2) reduction in parasite prevalence. Data were analysed using cluster-adjusted intention-to-treat (ITT) methods. The study was conducted from February 2020 to July 2021 and is registered with ClinicalTrials.gov (NCT04245033).

**Findings:**

Three rounds of DP dispensing covered 127 schools with more than 73,000 schoolchildren. IPTsc coverage reached 77% (range: 69–83%), 73% (64–81%), and 80% (76–85%) in August 2020, November 2020 and March 2021, respectively. DP was well tolerated. In the effectiveness evaluation, 1971 schoolchildren were enrolled in DP arm and 1781 in control arm. After one year, IPTsc reduced clinical malaria incidence by 41% overall (95% CI: 31–49; p < 0.001) and by 54% (95% CI: 44.2–62.6; p < 0.001) in high endemic strata (≥10% baseline prevalence). Parasite prevalence decreased by 81% overall (95% CI: 56.3–100; p < 0.001) ranging from 58% (95% CI: 10.9–100; p = 0.18) in low endemic to 83% (95% CI: 62.7–100; p < 0.001) in high endemic strata.

**Interpretation:**

Large scale implementation of IPTsc achieved impact comparable to randomised trials and was operationally feasible and well accepted by communities and teachers. This study sets a benchmark for school-based malaria chemoprevention in Tanzania and provides groundwork for national policy adoption.

**Funding:**

The 10.13039/100004417Global Fund, through the Ministry of Health, Tanzania, funded the study.

**Research in context**

**Evidence before this study**

Recent malaria control efforts create an epidemiological age shift in the malaria burden to older children (aged 5–15 years) making this age group more vulnerable for malaria. School aged children tend to comply less to malaria preventive & curative measures and substantially contribute to malaria transmission. We conducted a randomised controlled trial on Intermittent Preventive Treatment of malaria in schoolchildren (IPTsc) (Makenga et al., 2023, Lancet GH), we showed IPTsc highly effective with protective effect of 70% on malaria parasitaemia in school aged children. The study findings were consistent with several other studies analysed on systematic review (Cohee LM et al., 2020) showing a protective efficacy on malaria parasitaemia of 72% overall and that IPTsc was also an effective tool to reduce malaria related anaemia. IPTsc is potentially a very (cost-)effective tool with high treatment adherence minimising possible risk of drug resistance. Therefore, in 2023, WHO recommended IPTsc as a possible intervention.

**Added value of this study**

While substantial evidence on the impact of IPTsc has been generated in clinical trial settings, it has not translated into policy or practice. To address this gap, we conducted a large-scale implementation project sponsored by the Ministry of Health—the first-ever evaluation of IPTsc in a programmatic setting. The study demonstrated that IPTsc implementation is feasible, achieving coverage rates of up to 80% among more than 73,000 pupils, with over 165,748 doses of DP successfully administered by schoolteachers. Coverage closely mirrored routine school attendance, and a steady increase across successive rounds reflected strong community acceptance of the intervention. Implementation of IPTsc led to an 81% reduction in malaria prevalence—effectively lowering the parasite reservoir—in the intervention arm. It also demonstrated a protective effect of 41% against clinical malaria overall, increasing to 54% in high-transmission areas (≥10% malaria prevalence). These findings show that IPTsc can deliver outcomes comparable to those observed in randomised controlled trials. It is not only operationally feasible through school-based delivery but also highly accepted by both communities and educators.

**Implications of all the available evidence**

This project supported the national scale-up of IPTsc in Tanzania and provides critical evidence to inform WHO guidance and strengthen malaria control programmes in implementing the strategy with confidence. Following the results dissemination conference, the Tanzanian government recognised this study as a pilot that laid the foundation for policy development and established implementation standards for IPTsc in one-third of the country, specifically in high-endemic districts as defined by NMCP stratification.

Our findings also offer additional implementation evidence by demonstrating how children not reached through schools can be included through the engagement of community health workers at the village level. Malaria control programmes should consider IPTsc a key intervention opportunity, as recommended in the WHO's 2023 malaria guidelines.

Moreover, the observed impact of IPTsc across different transmission strata—defined by malaria prevalence—provides valuable insights for tailoring the implementation of WHO malaria guidelines to diverse epidemiological contexts.

## Introduction

The WHO Malaria report 2024, reported an estimated 263 million cases with an incidence of 60.4 cases per 1000 population at risk. The WHO African region, which is mostly comprised of sub-Saharan Africa, was home to 94% of malaria cases and 95% of malaria deaths globally. Tanzania is, with Nigeria, Democratic Republic of Congo, and Niger, amongst the four African countries accounting for over half of all malaria deaths worldwide.^1^

In malaria endemic areas, malaria is responsible for half of the mortality in school-aged children (5–15 years old).^2^ However, this age group harbours malaria parasites which are mostly asymptomatic,^2^, ^3^, ^4^, ^5^, ^6^ contributing to anaemia, poor cognitive development, and poor school performance.^2^^,^^6^, ^7^, ^8^ School-aged children also tend to less comply to malaria control preventive or curative measures^9^^,^^10^ and, so far, targeted malaria interventions ignore school-aged children, with main focus on pregnant women, through intermittent preventive treatment in pregnancy (IPTp), and children under five years of age—through seasonal malaria chemoprevention (SMC), perennial malaria chemoprevention (PMC), and distribution of insecticide-treated nets via antenatal and child health services. Additional preventive measures have been directed toward travellers.^1^ However, from a population health perspective, school-aged children contribute substantially as reservoir to onward malaria transmission in the population.^4^^,^^5^^,^^11^, ^12^, ^13^ Further, due to successful malaria control efforts, school-aged children have become increasingly more vulnerable as compared to those aged less than five years due to delayed acquisition of protective immunity^2^^,^^11^

In Tanzania, according to the National Malaria Control Programme (NMCP), malaria prevalence has declined substantially from 18.1% in 2008, meso-endemic level, to 8.1% in 2022, a hypo-endemic level. This necessitated a sub-national stratification to optimise cost-effective implementation of interventions. The stratification showed distinct variations in decline across and within regions and/or councils.^14^ Detailed analyses at council level, indicated that in moderate to high endemic councils, schoolchildren were the most affected age group. Based on malaria rapid diagnostic test (mRDT), the nationwide, school malaria survey conducted between 2014 and 2015 reported, the overall prevalence of malaria as 21.6%, ranging from 0 to 76.4% among councils.^4^ In this stratification, 73 (40%) councils were categorised as high endemic,^14^ meaning the existing malaria control tools are suboptimal^5^^,^^6^ (one size did not fit all), thus, calling for additional specific intervention tools.

Parasitic clearance using artemisinin-based combination therapies (ACTs) has shown to be effective in clearing or reducing gametocytes carriage post treatment^12^^,^^15^ and clinical trials have recommended use of ACTs for Intermittent preventive Treatment (IPTsc) in endemic settings where resistance to SP is high.^16^ A recent review on ACT impact on gametocytes argued that, if transmission is largely driven by asymptomatic individuals who do not seek treatment (especially school-aged children), then, the inclusion of these asymptomatically infected individuals in treatment campaigns may have a much larger impact on malaria transmission than the choice of ACT for first-line treatment.^12^

Following an impressive and successful IPTsc trial,^17^ the NMCP in Tanzania, endorsed the implementation of IPTsc with dihydroartemisinin–piperaquine (DP), an ACT alternative to the first line, in moderate to high endemic areas.^18^ Likewise, the WHO 2023 malaria guideline came up with a similar recommendation.^19^ In the present study, we evaluated the implementation of IPTsc using DP, delivered by schoolteachers, given three times a year, to provide evidence on the operational feasibility and IPTsc effectiveness on asymptomatic and symptomatic clinical malaria incidence at a highly endemic area in Tanzania. The study also provides an effective way of implementing IPTsc programme using both existing health and education systems.

## Methods

### Study design

This was an effectiveness-implementation hybrid trial to assess feasibility and effectiveness of IPTsc using DP against standard of care (control). The study methodology, challenges and mitigation have been described in details elsewhere.^20^

**Fig. 1:**
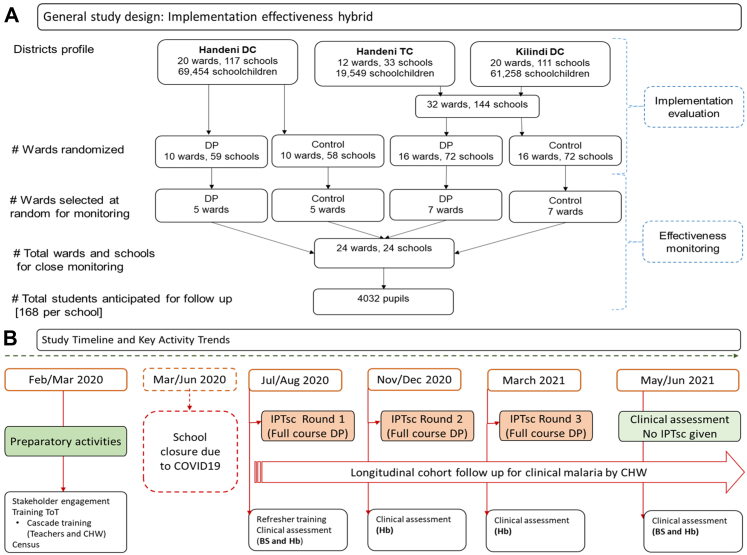
**Study setup for the IPTsc implementation evaluation and timelines for key activities in the three councils of Tanga region, northeast, Tanzania**.^20^ Legend: DC = District councill, TC = Town Council, DP = Dihydroartemisinin-Piperaquine, IPTsc round-a complete cycle of IPTsc drug deployment in all councils as per schedule-there were 3 rounds in total over a one year implementation, CHW = Community Health Worker, BS = Blood smear for microscopy, Hb = Haemoglobin.

For the implementation evaluation, wards within three councils (Handeni DC, Handeni TC, and Kilindi DC) served as randomisation units (clusters). Each ward was randomly assigned to either implement IPTsc or continue standard of care (control) (Fig. 1). DP was administered to all schoolchildren in class 1 to 7 at about four-month intervals, three times a year, in March, August and November, aligned with school curriculum schedule and local malaria transmission seasonality. To facilitate this, the study reimagined an existing school health programme for Neglected Tropical Diseases (NTD) control to implement IPTsc. Mixed methods were employed to assess the feasibility and acceptability of implementing IPTsc as part of a more comprehensive health package for schoolchildren.

A nested effectiveness assessment was included within the wider IPTsc implementation. This involved enrolling a subgroup of randomly selected schoolchildren who were monitored more intensively for malaria outcomes. This design allowed for detailed evaluation of IPTsc effectiveness while the broader implementation continued across all participating schools. In this, 12 wards were randomly selected in each study arm, one school per ward, and 168 pupils per school were chosen as representatives under close supervision, making a total of 4032 schoolchildren (Fig. 1). From selected representative pupils, finger pricked blood samples were collected for malaria microscopy (at baseline and last visit), and haemoglobin concentration (at each visit) to determine prevalence of malaria and anaemia, respectively. Monthly reports on absenteeism, clinical malaria episodes, reported adverse events were collected, in collaboration with schoolteachers, local health facility workers and the local community health workers (CHW).

### Participants implementation

In the intervention arm, all primary school pupils class 1–7, were eligible to receive IPTsc intervention.^20^ The primary school health teachers were trained for antimalarial (DP) drug administration, accountability and basic management of adverse events following drug administration. In addition, the community health workers, school health teachers, health workers from local health facilities and village administrators, were involved during surveys and in continued follow-up to capture adverse events, monitoring malaria episodes and school absenteeism. Study team comprising of interdisciplinary collaborations between the Ministry of Health (MoH), the Ministry of Education, Science, Technology and Vocational Training (MESTVT) and the President's Office Regional Administration and Local Government (PO-RALG) at national and local levels were set to supervise the IPTsc implementation.^20^

### Randomisation and masking for effectiveness assessment

Of the total 52 wards in the three councils, 26 wards were randomly selected to receive the IPTsc while the other half was not. This was done openly before council officials, using excel® (Microsoft, Seattle, US) generated random numbers. For the clinical effectiveness evaluation, 12 wards were randomly selected per study arm, and from each ward, one school was then randomly selected. In 2 out of 24 wards, a preselected school was replaced due to geographic or logistical challenges that would have made it unsafe or impractical for the study team to conduct reliable assessments with laboratory equipment. These schools were newer extensions serving pastoralist communities from distant areas, and in these cases, the main school was selected as the more feasible point of evaluation. In each selected school, a systematic sampling, balanced to gender and class, was conducted to obtain 168 schoolchildren who would be involved for IPTsc close monitoring after obtaining written informed consent from their parents. During clinical surveys, only specific study ID was used to label the samples hence laboratory team were blinded of study arm and identity of the participants.^20^

### Study procedures

#### IPTsc implementation

This study was designed to evaluate implementation of IPTsc as endorsed by the NMCP Tanzania, the study sponsor. From the outset, a comprehensive community engagement strategy was developed and implemented at national and local levels, involving political and technical leaders across respective tiers, as described elsewhere.^20^ This process was accompanied by training sessions to train trainers, who subsequently trained schoolteachers. The study medication, DP (D-Artepp®, Guilin Pharmaceutical Co, China), was procured and distributed through routine channels, delivered to each council by the Medical Stores Department (MSD). Council pharmacists, together with the study team, distributed the drugs to health facilities near schools in each intervention ward, from where schools collected the medications. Depending on the number of schoolchildren, at least seven teachers per school dispensed the drugs according to a drug dispensing instruction manual.^20^ This arrangement minimised disruption to routine school activities and ensured directly observed treatment for maximum compliance.

Schoolchildren in each intervention ward received DP three times per year (three rounds): August 2020 (Round 1), November 2020 (Round 2), and March 2021 (Round 3) (Fig. 1B). DP tablets (40 mg dihydroartemisinin and 320 mg piperaquine) were dosed according to each child's body weight, following manufacturer guidelines. Medication was administered with water approximately one to 2 h before lunch, although some children received the drug after meals due to operational considerations.^20^ A full course of DP is defined as completing the three-day regimen, with one daily dose administered under direct observation for three consecutive days. Children were observed for 30 min post-administration; if vomiting occurred during this period, a repeat dose was given once and recorded. Children who vomited after the second dose were withdrawn from further treatment. Children missing the first day's dose were given the opportunity to start on the second day. If the second-day dose was missed, community health workers followed up at the child's home to administer the dose within the same day or on the third day. Missing both the second and third doses was classified as an incomplete course, defined as receiving only one or two days of the three -day regimen.

#### IPTsc effectiveness

The evaluation commenced with a household census survey conducted between February and March 2020 (see Fig. 1), which collected socio-economic and demographic data through home visits of all children enrolled for effectiveness assessment. After a brief meeting and signing of informed consent forms, parents or guardians were interviewed at their homes. Observations related to malaria risk and prevention were also recorded, and GPS coordinates were taken for each household.^20^

Following school reopening after COVID-19 closures, enrolled children were clinically assessed at their schools during four scheduled visits spaced approximately every four months over 12 months (Fig. 1). Baseline clinical assessments took place in July–August 2020, prior to the first IPTsc round. Thick and thin blood smears were obtained prior to treatment at baseline, and month 12 from all participants. These were examined by expert microscopists blinded to participant identity to detect *Plasmodium* species and quantify parasite density. Haemoglobin concentration was measured at recruitment and during scheduled follow up visits using a haemoglobinometer (HemoCue AB®, Sweden). Health workers at local health facilities participated in documenting clinical events for enrolled children who sought care. During scheduled visits, any child presenting with symptoms suggestive of malaria (e.g., fever), regardless of study arm, was tested using malaria rapid diagnostic tests (mRDT) and treated per standard treatment guidelines. Two trained CHWs were assigned to each evaluation school. These CHWs diagnosed and treated uncomplicated malaria following national guidelines. Children who tested mRDT-negative or showed severe symptoms were referred to nearby health facilities, using a special referral form to prevent duplicate records. Parents and enrolled children were instructed to contact their CHW first if illness occurred, facilitating early diagnosis, treatment, and accurate documentation of all malaria episodes. All services were provided free of charge.

### Study outcome and endpoints

For the implementation evaluation, the primary outcome was the feasibility of implementing IPTsc in high-endemic regions, measured by coverage. The secondary outcome was the acceptability of IPTsc implementation among schoolchildren, parents/guardians, and teachers.

For the clinical effectiveness evaluation, the primary outcome was the (1) reduction in clinical malaria incidence, measured at month 12. and (2) the reduction in parasite prevalence from baseline (measured at month 12). Secondary outcomes included reduction in prevalence of anaemia, change in haemoglobin concentration, and the safety of DP.

### Case definitions and statistical analysis

IPTsc implementation coverage per round was defined as the number of schoolchildren who completed three day course of therapeutic-dose DP, divided by the total number of children enrolled in a given school or ward within the IPTsc arm.^20^

Malaria incidence in school-aged children from Handeni District Council (DC) was assumed to be 413 cases per 1000 population, based on Demographic Health Information System 2 (DHIS2) data, with an expected 40% reduction in the intervention arm compared with the control. Using an assumed correlation coefficient (k) of 0.2, 80% power, and a two-sided significance level of 0.05, five clusters per arm were required for Handeni DC. Similar parameters were applied to Kilindi DC and Handeni Town Council (TC), where baseline incidence was estimated at 183 cases per 1000; here, seven clusters per arm were needed (five from Kilindi DC and two from Handeni TC). Allowing for 20% attrition, the target enrolment was 4032 schoolchildren from 24 schools (1680 from Handeni DC, 672 from Handeni TC, and 1680 from Kilindi DC), which was calculated to yield approximately 3226 evaluable participants for analysis.^20^

Children enrolled in the sub study for close clinical monitoring were visited at home for demographic assessment. Principal component analysis (PCA) was used to categorise children and their households into different socioeconomic status (SES) groups. Household SES scores were grouped into three quantiles defined as low, medium and high. Variables included in the PCA were: housing characteristics (type and material of roof, walls, floor, and ceiling), type of toilet facility, presence of electricity, ownership of assets (radio, mobile phone, bicycle, motorbike, vehicle), number of sleeping rooms per household member, occupation of the household head, and the number of livestock and size of land owned by the family.^6^^,^^21^

Anaemia was defined using the World Health Organisation (WHO) age-specific cut off points for haemoglobin (<11.5 g/dL for children 6–< 12 years of age,< 12.0 g/dL for those 12–14 years of age, <13 g/dL and <12 g/dL for male and female children aged 15 years, respectively).^22^^,^^23^ The impact of IPTsc on anaemia at different clinical assessment points, relative to the control arm, was assessed using a linear mixed-effects model. The 2006 WHO Child Growth Standards was used to compute age- and sex-specific anthropometric indices. Specifically, we calculated Body mass index (BMI) -for-age z-scores (BAZ), height-for-age z-scores (HAZ), and weight-for-age z-scores (WAZ). The z-scores were generated in STATA with the “*zanthro*” package, which implements the WHO child growth standards for calculating anthropometric z-scores described elsewhere.^23^^,^^24^ Children were classified as underweight/wasted if they were less than two standard deviations (SD) below the reference mean.^6^^,^^23^

Clinical malaria cases were defined as febrile episodes confirmed by a positive mRDT and/or microscopy, in accordance with WHO diagnostic standards. Case detection was conducted by trained CHWs as part of passive surveillance (parents/guardians would first seek CHW before heading to hospital), with clinical management and documentation supported by local health facility staff. Protective efficacy (PE) was estimated using the formula:PE = (1 − RR) × 100, where RR is the risk ratio for the incidence of clinical malaria in the intervention vs control arm.^25^^,^^26^ Risk ratios were derived from Kaplan–Meier survival analyses comparing time to first malaria episode between groups. The 95% confidence intervals (CIs) were calculated, and a p-value of <0.05 was considered statistically significant when comparing the IPTsc arm with the control. Incidence rates of clinical malaria were calculated as the number of new cases divided by total person-time at risk, expressed per child-month.

Malaria parasite prevalence was calculated as the number of children with parasites detected on thick blood smear (regardless of species), divided by the total number of children tested.^23^ To estimate the impact of IPTsc on malaria prevalence, we used a difference-in-differences (DiD) approach. This method compares changes in malaria prevalence from baseline (Month 0) to endline (Month 12) between the intervention and control arms. It accounts for baseline imbalances and isolates the effect attributable to the intervention, as described elsewhere.^27^ Prevalence differences were first estimated within each arm, followed by between arm comparison using the DiD formula *[DiD = (Prevalence_M0 − Prevalence_M12)_Intervention − (Prevalence_M0 − Prevalence_M12)_Control].*

To account for the cluster-randomised design and repeated measures, linear mixed-effects model was used, treating schools as random effects to account for clustering and including random intercepts for individual participants. An intraclass correlation coefficient (ICC) of 0.2 was assumed based on prior IPT studies and design effect estimates.^17^ Analyses were stratified by baseline malaria prevalence, using a 10% threshold, in accordance with WHO guidance for IPTsc deployment in moderate-to high-transmission settings. Percentage reduction in malaria prevalence due to IPTsc was calculated from the DiD estimates using a linear mixed-effects model. Confidence intervals for the percentage reduction were derived using the delta method to approximate the variance.^28^ For reporting purposes, any confidence interval limits exceeding 100% were capped at 100%.

Data were analysed using both intention-to-treat (ITT) and modified ITT (M-ITT) frameworks. The ITT analysis included all enrolled children who participated in at least one follow up visit, while M-ITT analysis included children with complete data from both baseline (Month 0) and endline assessments (Month 12). Additional assessments were conducted at Months 4 and 8 to monitor trends over time (Fig. 1). All statistical analyses were conducted using STATA version 15 (StataCorp, College Station, TX, USA). Further details on supervision procedures and data collection for IPTsc coverage, clinical malaria episodes, and malaria parasitaemia are described elsewhere.^20^

### Ethics

The study obtained ethical approval from the Medical Research Coordinating Committee (MRCC, Tanzania) (NIMR/HQ/R.8a/Vol.IX/3291 and NIMR/HQ/R.8c/Vol.I/1652) and regulatory approval from the Tanzania Medicines and Medical Devices Authority (TMDA) (TMDA0019/CTR/0018/03). Informed consent was obtained from parents/guardian of schoolchildren involved in effectiveness evaluation. Programmatic implementation was permitted by respective authorities to the level of school parents committees. The study has been registered on ClinicalTrials.gov (NCT04245033) since January 20, 2020.

### Role of the funding source

The Global Fund, through the Ministry of Health, Tanzania, funded the study. Funding sources had no role in writing of the manuscript or the decision to submit it for publication. Authors were not precluded from accessing data in the study, and they accept responsibility to submit for publication.

## Results

### Implementation research

The IPTsc intervention was implemented by local government authorities and the NMCP in 26 of the 52 wards randomised to the intervention arm, 10 in Handeni DC, 6 in Handeni TC, and 10 in Kilindi DC. Across the three rounds, the programme reached 73,664 children in round 1, 68,294 in round 2, and 73,729 in round 3, from a total of 127 public primary schools (Table 1). The number of children per school reflected the population size within each ward.

**Table 1:** Schoolchildren enrolled in implementation study and dihydroartemisinin-piperaquine consumption in intervention arm in 3 councils in Tanga Region, Tanzania.

IPTsc Round	Council	Wards	School-children enrolled			DP (40 mg/320 mg) usage	
			Male	Female	All	No tabs used^a^	mean number of tabs per child (95% CI)
1	HANDENI DC	10	15,405	16,229	31,634	162,783	6.3 (5.9–6.6)
	HANDENI TC	6	5373	5242	10,615	56,601	6.5 (6.1–6.8)
	KILINDI DC	10	15,620	15,795	31,415	174,320	7.7 (5.6–9.7)
	**All Councils**	**26**	**36,398**	**37,266**	**73,664**	**393,704**	**6.9 (6.1**–**7.6)**
2	HANDENI DC	10	14,414	14,883	29,297	150,052	6.3 (6.0–6.6)
	HANDENI TC	6	5009	4877	9886	46,484	5.9 (5.1–6.7)
	KILINDI DC	10	14,479	14,632	29,111	119,944	6.5 (6.0–7.0)
	**All Councils**	**26**	**33,902**	**34,392**	**68,294**	**316,480**	**6.3 (6.0**–**6.5)**
3	HANDENI DC	10	15,673	16,585	32,258	167,933	6.1 (5.8–6.4)
	HANDENI TC	6	5519	5618	11,137	51,705	6.1 (5.5–6.7)
	KILINDI DC	10	14,925	15,409	30,334	143,167	6.2 (6.0–6.4)
	**All Councils**	**26**	**36,117**	**37,612**	**73,729**	**362,805**	**6.1 (6.0**–**6.3)**

a This includes those dropped or damaged or used on re-dosing.

Round 1 was conducted from 3 to 5 August 2020, following the long rainy season; round 2 took place from 16 to 20 November 2020 during the short rainy season; and round 3 occurred from 1 to 5 March 2021, before the seasonal malaria peak and onset of the long rains. The mean number of DP (40 mg/320 mg) tablets dispensed per child declined from 6.9 (95% CI: 6.1–7.6) in round 1 to 6.1 (95% CI: 6.1–6.3) in round 3. This reduction reflected improved drug accountability, reduced wastage, and increased coverage, as a higher proportion of children completed the full three-day DP regimen over time.

#### Coverage and safety

**Fig. 2:**
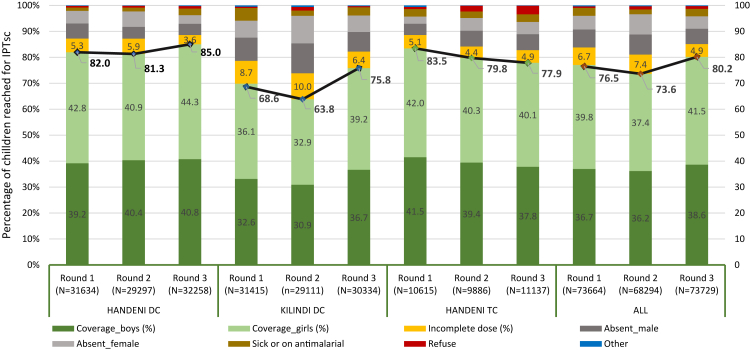
**IPTsc coverage: Proportion of schoolchildren completing dihydroartemisinin-piperaquine dose per IPTsc round and per council in an implementation study in 3 councils in Tanga Region, Tanzania**.

The overall coverage (the proportion of participants completing the full three-day DP regimen) in the first IPTsc round was 76.5%, with council-specific coverage of 82% (range 78.0–93.4%) in Handeni DC, 84% (75.7–89.7%) in Handeni TC, and 69% (56.1–83.9%) in Kilindi DC (Fig. 2; Supplementary Figures S1, S4–S7). In the second round, which coincided with the graduation and departure of Class 7 pupils, overall coverage declined to 73.6% (Handeni DC: 81% [69.1–95.5%], Handeni TC: 80% [59.4–88.9%], Kilindi DC: 64% [41.8–79.9%]). Coverage improved in the third round, reaching 80% overall, with council-specific rates of 85% (74.2–97.6%), 77.9% (74.3–83.5%), and 75.8% (68.6–89.2%) for Handeni DC, Handeni TC, and Kilindi DC, respectively. Across all councils, coverage patterns reflected routine school attendance. In rounds 1 and 3, 12.1% and 10.7% of pupils, respectively, were absent and untraceable (grey bars, Fig. 2); 3.0% and 2.8% were reported sick (brown bars); and 0.6% and 1.1% refused medication (red bars). The proportion of children receiving an incomplete course—defined as one or two days of the three-day regimen—declined from 6.7% in round 1 to 4.9% in round 3, suggesting improved follow-up by CHWs.

Across all three IPTsc rounds, 165,748 DP doses were administered, with 49 adverse drug reaction (ADR) reports (0.03% of doses). These were distributed as follows: Handeni TC (26), Handeni DC (5), and Kilindi DC (18) (Supplementary Figure S1). Nearly half (49%, n = 24) occurred in the first round. Most ADRs (87.8%, n = 43) were non-serious. Of the six serious adverse events (SAEs), five involved headache, vomiting, and general weakness; all resolved following appropriate management. One SAE was fatal due to suspected herbal intoxication, considered unrelated to IPTsc; the child had participated in all three rounds without prior side effects. All ADRs and SAEs were reviewed by the study supervision team in collaboration with the national regulatory authority's pharmacovigilance unit.

### IPTsc effectiveness evaluation

#### Enrolment and follow up

**Fig. 3:**
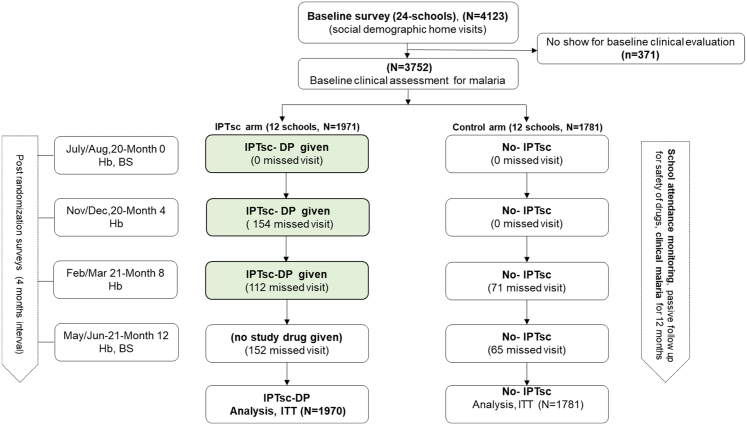
**Study flow chart for the effectiveness evaluation in an IPTsc implementation research study in north-east, Tanzania**. Legend: BS = Blood Smear for microscopy, DP = Dihydroartemisinin-Piperaquine, Hb = Haemoglobin, ITT = Intention to Treat.

A census survey conducted in February 2020 identified 922 households linked to the selected schools. These households included 4123 children aged 5–15 years, of whom 50.5% (n = 2082) were female. At the baseline school visit, 371 pupils were absent, leaving 3752 children available for clinical assessment (1971 in the IPTsc arm and 1781 in the control arm; Fig. 3).

#### Baseline characteristics

The mean age of participants was 10.3 years (SD 2.5), with 65% reporting a history of malaria in the previous month. Bed net ownership was 47.4%, while reported usage was 27.1% (Supplementary Table S1). The overall baseline malaria prevalence was 36.5% (n = 1345), significantly higher in the DP arm (38.6%, 748/1939) than in the control arm (34.1%, 597/1749; p = 0.005). Malaria prevalence varied substantially across councils, with Handeni DC exhibiting the highest prevalence at 58.4% (n = 925), including 61.9% (n = 493) in the DP arm; all clusters in this council were classified as high-transmission strata. Handeni TC had the lowest prevalence at 19.6% (n = 98), again higher in the DP arm (26%, n = 72) than in the control arm (12%, n = 26), with three of four clusters designated as high strata. Kilindi DC recorded a prevalence of 20.1% (n = 322), with 21.1% (n = 183) in the DP arm and 18.9% (n = 139) in the control arm; half of its clusters were classified as low strata. The mean *Plasmodium falciparum* parasite density was 6556.5/μL (Supplementary Table S1), with a lower mean density of 4683/μL observed among asymptomatic cases. Fever prevalence was 11% (n = 421), of whom 60% (n = 252) tested positive for malaria. Anaemia prevalence was 36.3% (n = 1363), highest in Handeni DC at 52.8% (n = 851) and lowest in Handeni TC at 22.1% (n = 113) (Supplementary Table S1).

#### Impact of IPTsc on clinical malaria

During follow-up, a total of 760 new clinical malaria cases were recorded at 12 months, with 461 cases in the control arm and 299 cases in the intervention (DP) arm. After 2 rounds of IPTsc (assessment at 8 months), the malaria incidence rate (IR) in the control arm was 0.04 cases per child-month (95% CI: 0.04–0.05) compared with 0.02 cases per child-month (95% CI: 0.02–0.03) in the DP arm, corresponding to an overall 47% protective effect (PE, 95% CI: 32.2–59.4) and a number needed to treat (NNT) of 47 (95% CI: 34–75) to prevent one malaria case (Table 2A & B). The effect was more pronounced in high-transmission strata (PE = 56%, 95% CI: 36.3–69.5; NNT = 53), with incidence falling from 0.03 to 0.01 cases per child-month, compared with a smaller and borderline significant reduction in low-transmission strata (PE = 30%, 95% CI: −1.4–51.4).

**Table 2:** Clinical Malaria trends among participants of an IPTsc implementation study in north-eastern Tanzania, based on survival model estimates.

A: Summary on person-time, failures and incident rate per study arm and strata					
IPTsc round	Study arm	Strata	Person-time (child months)	New malaria cases	Incidence rate (cases per child-month 95% CI)
2 Rounds (Assessment at 8 months)	Control	All	3949	179	0.04 (0.04–0.05)
		High strata	2784	95	0.03 (0.03–0.04)
		Low strata	1165	84	0.07 (0.06–0.09)
	DP	All	4022	96	0.02 (0.02–0.03)
		High strata	3038	46	0.01 (0.01–0.02)
		Low strata	985	50	0.05 (0.04–0.07)
3 Rounds (Assessment at 12 months)	Control	All	13,080	461	0.04 (0.03–0.04)
		High strata	9587	294	0.03 (0.03–0.03)
		Low strata	3493	167	0.05 (0.04–0.06)
	DP	All	14,387	299	0.02 (0.02–0.02)
		High strata	10,981	154	0.01 (0.01–0.02)
		Low strata	3407	145	0.04 (0.04–0.05)

B: Summary on protective effect against clinical malaria at different rounds during the intervention period						
Comparison	Comparison	Strata	risk ratio (95% CI)	PE (95% CI)	NNT (95% CI)	p-value
2 Rounds (Assessment at 8 months)	DP vs Control	All	0.53 (0.41–0.68)	47 (32.2–59.4)	47 (34–75)	**<0.0001**
		High strata	0.44 (0.31–0.64)	56 (36.3–69.5)	53 (37–92)	**<0.0001**
		Low strata	0.70 (0.49–1.01)	30 (48.6–51.4)	47 (24–2234)	**0.0241**
3 Rounds (Assessment at 12 months)	DP vs Control	All	0.59 (0.51–0.68)	41 (31.3–48.9)	70 (55–97)	**<0.0001**
		High strata	0.46 (0.37–0.56)	54 (44.2–62.6)	60 (48–80)	**<0.0001**
		Low strata	0.89 (0.71–1.12)	11 (−11.9 to 29.2)	191 (65–209)	0.1531

Bolded p values indicate statistical significance (p < 0.05).

Legend: CI = Confidence Interval; DP = Dihydroartemisinin–Piperaquine; PE = Protective Effect (% reduction in incidence rate compared with control); NNT = Number Needed to Treat (children treated for one year to prevent one malaria case); IPTsc = Intermittent Preventive Treatment in schoolchildren.

After 3 rounds of IPTsc (assessment at 12 months), IRs remained about twice as high in the control arm (0.04 cases per child-month, 95% CI: 0.03–0.04) as in the DP arm (0.02 cases per child-month, 95% CI: 0.02–0.02), yielding an overall PE of 41% (95% CI: 31.3–48.9) and an NNT of 70 (95% CI: 55–97). The benefit persisted in high-transmission strata (PE = 54%, 95% CI: 44.2–62.6; NNT = 60) but was not statistically significant in low-transmission strata (PE = 11%, 95% CI: −11.9 to 29.2). Survival analysis of time to first clinical malaria episode is presented in Supplementary Figure S3.

#### Impact of IPTsc on asymptomatic malaria parasitaemia

Malaria parasitaemia reduced to 31% (534/1718) and 21% (377/1818) in control and intervention arms, respectively (Table 3). The intervention group had a cluster adjusted malaria prevalence difference (at *icc* = 0.2) from baseline of 17.8% (95% CI: 14.9–34.2; p = 0.04) compared to 3% in control arm (95% CI: −14.2 to 20.3; p = 0.73). In high strata areas, the difference was more pronounced with 21% (95% CI: 1.4–41.2; p = 0.04) in intervention compared to 3.9% (95% CI: −17.2 to 24.9, p = 0.72) in control arm. In the cluster adjusted linear mixed model, the malaria prevalence difference from differences was −14.5% (95% CI: −22.0 to 6.9, p < 0.001) and −13.9% (95% CI: −21.4 to 6.4; p < 0.001), respectively on ITT and mITT analysis (Table 3A). The cluster adjusted difference in differences (DiD) showed a protective efficacy of the IPTsc intervention against malaria parasitaemia prevalence of 81% (95% CI: 56.3–100; p < 001) ranging from 58% (95% CI: 10.9–100; p = 0.18) in low strata to 83% (95% CI: 62.7–100; p < 0.001) in high strata (Table 3A).

**Table 3:** Impact of IPTsc on asymptomatic malaria prevalence reduction and on anaemia among participants in an implementation study in north-eastern Tanzania.

A. Impact of IPTsc on Asymptomatic Malaria Prevalence Reduction (Adjusted by Cluster)															
Analysis type	DP						Control						Reduction by difference in difference (DP vs Control)		
	Baseline (M0)		Month 12		Difference (M0-M12)		Baseline (M0)		Month 12		Difference (M0-M12)		Cluster adjusted prevalence change (DiD) (95% CI)	p-value	% Reduction by IPTsc (95% CI)
	N	Prev %	N	Prev %	Diff % (95% CI)	p-value	N	Prev %	N	Prev %	Diff % (95% CI)	p-value			
ITT (unadjusted-crude)	1939	38.6	1818	20.7	17.8 (15.0–20.7)	**<0.001**	1749	34.1	1718	31.1	3 (−0.0 to 6.2)	0.055	−14.5 (−17.9 to 11.0)	**<0.001**	81.5 (63.3–99.7)
ITT (k = 12)	1939	38.6	1818	20.7	17.8 (14.9–34.2)	**0.036**	1749	34.1	1718	31.1	3.0 (−14.2 to 20.3)	0.7293	−14.5 (−22.0 to 6.9)	**<0.001**	81.5 (56.3–100)
Modified ITT (k = 12)	1625	37.8	1647	20.8	17.0 (0.5–33.3)	**0.046**	1472	33.9	1498	30.8	3.0 (−14.3 to 20.4)	0.73	−13.9 (−21.4 to 6.4)	**<0.001**	81.8 (57.5–100)
ITT-high strata (k = 9)	1504	48.5	1384	27.2	21.3 (1.4–41.2)	**0.041**	1316	44.1	1306	40.3	3.9 (−17.2 to 24.9)	0.7192	−17.3 (−25.8–8.8)	**<0.001**	81.2 (61.3–100)
M-ITT-high strata (k = 9)	1269	47.1	1286	26.6	20.5 (0.6–40.4)	**0.048**	1118	43.6	1141	40.0	3.6 (−17.6 to 24.8)	0.74	−17.0 (−25.5 to 8.5)	**<0.001**	82.9 (62.7–100)
ITT-low strata (k = 3)	435	4.4	434	0.2	4.1 (−6.7 to 15.0)	0.458	433	3.7	412	1.9	1.7 (−10.3 to 13.8)	0.78	−2.4 (−5.8 to 1.0)	0.18	58.5 (10.9–100)
M-ITT-low strata (k = 3)	356	4.5	361	0.3	4.2 (−6.8 to 15.2)	0.457	354	3.4	357	1.7	1.7 (−9.7 to 13.2)	0.77	−2.5 (−6.3 to 1.3)	0.20	59.5 (6.3–100)
Avg cluster size, CV -ITT	161.6, 0.14		151.5, 0.09				145.7, 0.18		143.2, 0.18						
Avg cluster size, CV- M-ITT	135.4, 0.13		137.2, 0.13				122.7, 0.21		124.8, 0.21						

B. Change in Anaemia from baseline at different follow-up points.									
Time point from baseline	Analysis	Study arms, Number tested (mean Hb g/dl)				Anaemia prevalence change from baseline (DP vs Control)			
		DP		Control		Adjusted∗ prevalence change (95% CI)	p-value	Cluster adjusted prevalence change (95% CI)	p-value
Intervention year		n/N	%	n/N	%				
Jul/Aug 2020 Month 0 (baseline)	ITT	736/1970	37.4	627/1781	35.2	NA (NA)	NA	NA (NA)	NA
	Modified ITT	615/1647	37.3	958/1498	36.1	NA (NA)	NA	NA (NA)	NA
	High strata (ITT)	657/1528	43.0	572/1341	42.7	NA (NA)	NA	NA (NA)	NA
	Low strata (ITT)	79/442	17.9	55/440	12.5	NA (NA)	NA	NA (NA)	NA
Nov/Dec 2020 Month 4	ITT	679/1816	37.4	659/1783	37.0	−0.93 (−4.5 to 2.6)	0.605	−0.93 (−12.0 to 10.1)	0.869
	Modified ITT	557/1453	38.3	510/1370	37.2	−0.07 (−3.9 to 3.8)	0.973	0.07 (−11.6 to 11.6)	0.991
	High strata (ITT)	588/1377	42.7	614/1352	45.4	−2.61 (−6.9 to 1.7)	0.231	−2.61 (−16.9 to 11.6)	0.718
	Low strata (ITT)	91/439	20.7	45/431	10.4	5.51 (−0.0 to 11.1)	**0.051**	5.51 (−4.6 to 15.6)	0.284
Feb/Mar 2021 Month 8	ITT	725/1858	39.0	703/1712	41.1	−4.44 (−8.0 to 0.9)	**0.014**	−4.44 (−10.6 to 1.7)	0.159
	Modified ITT	582/1504	38.7	568/1361	41.7	−4.73 (−8.6 to 0.9)	**0.016**	−4.73 (−11.2 to 1.7)	0.151
	High strata (ITT)	642/1427	50.0	644/1302	49.5	−5.31 (−9.6 to 1.0)	**0.015**	−5.31 (−13.0 to 2.4)	0.176
	Low strata (ITT)	83/431	19.3	59/410	14.4	−0.66 (−6.3 to 4.9)	0.816	−0.66 (−7.5 to 6.2)	0.849
May/Jun 2021 Month 12	ITT	491/1818	27.0	536/1718	31.2	−5.82 (−9.4 to 2.3)	**0.001**	−5.82 (−15.0 to 3.4)	0.215
	Modified ITT	450/1647	27.3	463/1498	30.9	−4.88 (−8.6 to 1.1)	**0.011**	−4.88 (−14.2 to 4.4)	0.304
	High strata (ITT)	434/1384	31.4	471/1306	36.1	−4.99 (−9.3 to 0.7)	**0.023**	−4.99 (−16.5 to 6.5)	0.394
	Low strata (ITT)	57/434	13.1	65/412	15.8	−7.36 (−13.0 to 1.8)	**0.010**	−7.36 (−13.0 to 1.7)	**0.011**

Bolded p values indicate statistical significance (p < 0.05).

Legend: Adjusted∗ for individual child random-effects parameters, ITT = Intention to Treat, ICC = interclass correlation estimated following xtmixed model in ITT; Within- and between-group comparisons of malaria prevalence change from baseline (Month 0) to Month 12, adjusted for clustering by school (intraclass correlation coefficient = 0.2). k: number of clusters (schools) per study arm. ITT: Intention to Treat. M-ITT: Modified Intention to Treat. DiD: Difference-in-differences in prevalence, estimated using a linear mixed model. Confidence intervals for the percentage reduction were derived using the delta method to approximate the variance.^28^

#### Impact of IPTsc on anaemia

In the course of one year, anaemia prevalence declined from 37.4% (736/1970) to 27.0% (491/1818) in the intervention arm, and from 35.2% (627/1781) to 31.2% (536/1718) in the control arm. Using a DiD approach, the unadjusted reduction in anaemia prevalence attributable to IPTsc was 58% (95% CI: 22–90%, p < 0.001). When analysed using a linear mixed model adjusting for individual-level covariates, the intervention arm (DP) showed a significant 6% absolute reduction in anaemia prevalence compared to the control arm (95% CI: 2.3–9.4%, p < 0.001). However, this significance was not retained after adjusting for clustering by school, except in the low-transmission strata, where the IPTsc arm showed a 7.4% reduction in anaemia prevalence at Month 12 (95% CI: −13.0 to −1.7, p = 0.011). Additional stratified and cluster-adjusted estimates are provided in Table 3B. In the course of one year (month 12) of intervention implementation, in a linear mixed model, there were changes from baseline in haemoglobin levels (Hb) among participants in the intervention arm compared to those in control arm (0.31 g/dL, 95% CI: 0.2–0.4, p < 0.001), this was on individual participant but fell short of significance on cluster adjustment (Supplementary Table S3). Corresponding trends in mean haemoglobin levels (Hb) across different follow-up periods are presented in Supplementary Table S3 and Figure S2.

## Discussion

This pilot study evaluated the feasibility and acceptability of a programmatic implementation and related effectiveness of IPTsc in Tanzania. The study was conducted following a successful IPTsc clinical trial conducted in Muheza Tanga Region.^17^ IPTsc implementation was feasible with high coverages reaching 80% of the >73,000 pupils reached in the intervention arm. Coverage was synonymous to routine school attendance. An incremental trend in coverage at each round from first to the third pictured good acceptability of the IPTsc intervention in the communities. A subgroup (“nested effectiveness assessment”) within the wider IPTsc implementation, consisting of randomly selected children monitored more closely, showed an 81% reduction in malaria prevalence and a protective effect of 41% on clinical malaria incidence (54% in high strata). The study drugs were safe and well tolerated among schoolchildren. Thus, IPTsc may be an extremely useful tool to reduce disease burden and control transmission in endemic communities where school aged children remain vulnerable and act as reservoirs of infection. It led Tanzania to install an IPTsc country policy and laid standards for IPTs implementation in Tanzania and hopefully also elsewhere with similar circumstances. Intervention such as test and treat tested elsewhere showed no impact in school aged children.^29^

Our findings are consistent to other similar studies on effectiveness of IPTsc.^30^ Given the impact on IPTsc on reducing malaria parasitaemia prevalence and on clinical malaria incidence, combined with the high correlation of asymptomatic malaria prevalence between school-aged children and other vulnerable populations in the community,^5^ it is likely that a community-level effect would be observed. Studies in Uganda showed a high impact of IPTsc on communities where IPTsc was implemented,^31^ proving a phenomenon that IPTsc shrinks the malaria reservoir in the moderate and high endemic settings. An analysis on health economics of IPTsc is ongoing, however, elsewhere, this intervention has already shown to be cost effective and relatively simple to implement.^32^ The study hybrid design has enabled us to evaluate both implementation and effectiveness of IPTsc in Tanzania. The design gives a clear picture on feasibility of IPTsc implementation and the imagined means to simplify it for easier operationalisation by schoolteachers without disturbing the routine school curriculum activities. It is thus worth scaling up the programme to moderate and high endemic areas of the sub-Saharan Africa. On analysis we stratified according to WHO's guideline 2023 recommending IPTsc in areas where prevalence is greater or equal to 10%.^19^ We have indeed shown the high impact on higher strata (54%) than in lower strata (11%). Thus, the study has added more evidence for resolving uncertainties regarding effectiveness as well as reaching out school aged children in the communities.^20^ We have described how feasible IPTsc can be implementable and how communities can contribute to successful implementation of an intervention they see has a tangible impact.

Absenteeism has been the main reason for missing IPTsc doses. In this study, 11% of schoolchildren were unreached for IPTsc due to absenteeism, which was more commonly observed among boys than girls. Apart from absenteeism, another reason for missing dose was being sick or on antimalarial treatment during the IPTsc delivery week (3%). Refusal to take the medication was a rare reason for not receiving IPTsc (<1% overall). However, refusal was somewhat prominent in town council (3% by round 3) compared to rural councils (<1% by round 3). This pattern was expected since the risk and thus perceived risk, of malaria is quite low in towns compared to rural areas such as Handeni DC. Also the access to health facilities and medication for treatment is easier in town than in rural areas as noted elsewhere.^4^^,^^33^ CHWs reached out to those who missed the dose on IPTsc administration day, yet around 10% could not be reached to complete the dosing. This was mostly due to geographical terrain in hard-to-reach areas (e.g., Kilindi DC), where depending on weather of the day, children may or may not attend school and or conditions such as timing of the day may not be suitable for CHWs visit. Thus, a further community or parents/guardian support is needed to ensure IPTsc reaches all intended targets to ensure equity as required by the WHO guideline.^19^ In a similar thinking, though nowadays there are high primary school enrolment,^34^ there is still a high need to reach school aged children not recruited to schools, these may be in orphanage centres, street children, or children residing in the communities due to various social circumstances. Thus, CHWs will be needed to reach these groups in collaboration with respective departments at the ministry of social welfare.

Regarding the implementation aspect of this study, challenges and mitigation to inform policy development has been explained previously.^20^ Given the strategy designed to reach communities (parents, guardians or village members), the study provided important foundations for program scale up, as the involved officials would be capable of continuing implementation and acting as trainers for scaling up in other similar councils. The average number of DP tablets used per child (six tablets) served as a logistical baseline for drug distribution and will be useful for future procurement planning. However, this applies specifically to the DP drug used in this study. A different dosing algorithm would need to be developed if another drug is used for IPTsc, especially in a different population setting. The decreased drug wastage reflected in Table 1 may be partially explained by fewer children requiring redosing as they become accustomed to the drugs, reducing fear of adverse events. This is supported by our safety data, which show that most adverse events, such as mild vomiting and headaches, were more common in the first round than in subsequent rounds. Additionally, improved accountability mechanisms significantly contributed to reducing losses. Teachers were required to provide explanations for any missing tablets and reconcile the drugs received with the health facility pharmacist. Since DP administration was weight based, obtaining weighing scales for all schools in future implementation may be challenging. One option could be utilising facilities at a nearby health facilities for weighing students a week prior to IPTsc administration (an option we trialled with NMCP in Tanzania's northwest region), although this could be time consuming and may lack sustainability. Therefore, an ideal situation would be to develop a weight, height or age-based algorithm to facilitate easier DP administration, even in the absence of weighing scales.

In Tanzania, already the NMCP implements school net programme SNP especially in the southern and northern regions of Tanzania. Also there is a national school health programme (NSHP), that combines schistosomiasis and soil transmitted helminths (STH) control package under integrated neglected tropical diseases (NTD) programme.^20^ Therefore, there is potential for future integration to incorporate malaria interventions through school health programme.^20^^,^^35^ Given, the current government efforts of free primary education, there is high primary school enrolment rate in the country. Thus, schools act as a platform for health interventions delivery (e.g., SNP, awareness campaigns e.g., water sanitation and hygiene (WASH), deworming, iron-folic acid, nutrients supplementation, and boost-immunisation).

This study has several limitations inherent to its cluster randomised design. Defining clusters at the ward level—an administrative unit larger than villages, resulted in some baseline heterogeneity despite stratified randomisation by district malaria incidence. Differences in factors such as bed net usage, socioeconomic status, and fever prevalence between groups reflect the inclusion of semi-urban clusters alongside rural wards, leading to unavoidable imbalance. To mitigate potential confounding, we applied a Difference-in-Differences analytical approach with linear mixed models adjusted for cluster effects and stratified analyses by baseline malaria prevalence. While a baseline cross-sectional survey could have minimised randomisation imbalance, the trial's pragmatic design prioritised feasibility using district-level data for powering and stratification. We further addressed cluster variability by analysing outcomes across multiple randomisation levels (council, school, individual), with results remaining stable. The convenience selection of two schools out of 24 may limit generalisability but was necessary to ensure study team safety and data collection feasibility, and systematic selection criteria were maintained to preserve external validity. As an open-label trial, investigators were aware of treatment allocation, though laboratory personnel were blinded, and clinical assessments occurred prior to drug administration to minimise assessment bias. In low-prevalence strata, the intervention's effect did not reach statistical significance, likely due to limited baseline malaria prevalence, though the protective efficacy observed remains relevant. Future studies should explore the intervention's capacity to control the malaria reservoir in such settings. Despite these limitations, the rigorous analytical methods applied and pragmatic adaptations support confidence in the robustness and applicability of our findings.

Schoolchildren will continue to be vulnerable and act as substantial reservoirs of malaria transmission^11^ in any endemic community unless an intervention, i.e., IPTsc, geared to shrink the reservoir is deployed in these communities. Other available interventions such as bednets have shown to be less effective in school aged children. Only chemoprevention (IPTsc) is an intervention with direct impact to reduce malaria parasitaemia and malaria related morbidities so far. The clinical trial results in Tanzania^17^ and elsewhere are now shown via this implementation research^20^ how IPTsc is feasible and effective. We thus, highly recommend malaria endemic countries to implement IPTsc, now with high certainty that it works.

## Contributors

GMa, BPM, VB, FM, JPVg and JPAL conceptualised the idea. GMa wrote the manuscript. GMa, MDS, DPC, MC, HB, FF, DTRM, EL and AN contributed to tools generation. GMa, MDS, JPVg and JPAL did the approval procedures of the study by ethical committees and regulatory authorities. GMa, BPM, FF, VB, DTRM, DPC, EL, HB, JPAL, GMt, AL, FC, and JPVg accessed and verified the accessed and verified the underlying data, and contributed to formal analysis. GMa, MDS, AL,FF, MC, FC, DPC, AD,VB, DTRM, GMt, CM, SG, MS, MK, SJN, PMH, JRM, HS, AM, SA, JBT, ADM, AD, SGT, EK and JPAL implemented the study protocol in the field. MC, SL, JPVg, AMo, and JPAL supervised the conduct of the study. All authors reviewed and approved the submitted manuscript.

## Data sharing statement

All relevant data are within the manuscript and its Supporting Information files. However, in case of further details, data is available from the corresponding author on reasonable request and approval from the collaborating institutions and signing the data transfer agreement (DTA) from the National Institute for Medical Research (NIMR), Tanzania.

## Declaration of interests

Authors declare no competing interest.

## References

[1] Organization WH (2024).

[2] Nankabirwa J., Brooker S.J., Clarke S.E. (2014). Malaria in school-age children in Africa: an increasingly important challenge. Trop Med Int Health.

[3] Nankabirwa J.I., Wandera B., Amuge P. (2014). Impact of intermittent preventive treatment with dihydroartemisinin-piperaquine on malaria in Ugandan schoolchildren : a. Clin Infect Dis.

[4] Chacky F., Runge M., Rumisha S.F. (2018). Nationwide school malaria parasitaemia survey in public primary schools, the United Republic of Tanzania. Malar J.

[5] Makenga, Menon S., Baraka V. (2020). Prevalence of malaria parasitaemia in pregnant women and school aged children living in similar endemic setting of sub Saharan Africa: a systematic review and meta-analysis. Parasite Epidemiol Control.

[6] Makenga G., Baraka V., Francis F. (2022). Attributable risk factors for asymptomatic malaria and anaemia and their association with cognitive and psychomotor functions in schoolchildren of north-eastern Tanzania. PLoS One.

[7] Clarke S.E., Rouhani S., Diarra S. (2017). Impact of a malaria intervention package in schools on plasmodium infection, anaemia and cognitive function in schoolchildren in Mali: a pragmatic cluster-randomised trial. BMJ Glob Health.

[8] Halliday K.E., Karanja P., Turner E.L. (2012). Plasmodium falciparum , anaemia and cognitive and educational performance among school children in an area of moderate malaria transmission : baseline results of a cluster randomized trial on the coast of Kenya. Trop Med Int Health.

[9] Kihwele F., Gavana T., Makungu C. (2023). Exploring activities and behaviours potentially increases school-age children's vulnerability to malaria infections in south-eastern Tanzania. Malar J.

[10] Buchwald A.G., Walldorf J.A., Cohee L.M. (2016). Bed net use among school-aged children after a universal bed net campaign in Malawi. Malar J.

[11] Markwalter C.F., Lapp Z., Abel L. (2024). Mosquito and human characteristics influence natural anopheline biting behavior and Plasmodium falciparum transmission. medRxiv.

[12] WWARN Gametocyte Study group (2016). Gametocyte carriage in uncomplicated Plasmodium falciparum malaria following treatment with artemisinin combination therapy: a systematic review and meta- analysis of individual patient data. BMC Med.

[13] Walldorf J.A., Cohee L.M., Coalson J.E. (2015). School-age children are a reservoir of malaria infection in Malawi. PLoS One.

[14] Thawer S.G., Chacky F., Runge M. (2020). Sub-national stratification of malaria risk in mainland Tanzania: a simplified assembly of survey and routine data. Malar J.

[15] Bousema J.T., Schneider P., Gouagna L.C. (2006).

[16] Nankabirwa J., Cundill B., Clarke S. (2010). Efficacy, safety, and tolerability of three regimens for prevention of malaria: a randomized, Placebo- controlled trial in Ugandan schoolchildren. PLoS One.

[17] Makenga G., Baraka V., Francis F. (2023). Effectiveness and safety of intermittent preventive treatment with dihydroartemisinin-piperaquine or artesunate-amodiaquine for reducing malaria and related morbidities in schoolchildren in Tanzania: a randomised controlled trial. Lancet Global Health.

[18] NMCP (2020). National guidelines for malaria diagnosis, treatment and preventive therapies. Minist Health Tanznaia.

[19] World Health Organization (2023). WHO guidelines for malaria - 14 March 2023. http://apps.who.int/bookorders.

[20] Makenga G., Seth M.D., Baraka V. (2023). Implementation research of a cluster randomized trial evaluating the implementation and effectiveness of intermittent preventive treatment for malaria using dihydroartemisinin-piperaquine on reducing malaria burden in school-aged children in Tanzania: met. Malar J.

[21] Mmbando B.P., Segeja M.D., Msangeni H.A. (2009). Epidemiology of malaria in an area prepared for clinical trials in Korogwe, north-eastern Tanzania. Malar J.

[22] WHO (2019). http://https//www.who.int/vmnis/indicators/haemoglobin.pdf.

[23] Nankabirwa J., Wandera B., Kiwanuka N., Staedke S.G., Kamya M.R., Brooker S.J. (2013). Asymptomatic plasmodium infection and cognition among primary schoolchildren in a high malaria transmission setting in Uganda. Am J Trop Med Hyg.

[24] Vidmar S., Carlin J., Hesketh K., Cole T. (2004). Standardizing anthropometric measures in children and adolescents with new functions for Egen. Stata J.

[25] Matangila J.R., Doua J.Y., Mitashi P. (2017). International journal of antimicrobial agents efficacy and safety of intermittent preventive treatment in schoolchildren with sulfadoxine/pyrimethamine (SP) and SP plus piperaquine in democratic Republic of the Congo : a randomised controlled trial. Int J Antimicrob Agents.

[26] Clarke S.E., Jukes M.C., Njagi J.K. (2008). Effect of intermittent preventive treatment of malaria on health and education in school children: a cluster-randomised, double-blind, placebo-controlled trial. Lancet.

[27] Dimick J.B., Ryan A.M. (2014). Methods for evaluating changes in health care policy: the difference-in-differences approach. JAMA.

[28] Baker A., Callaway B., Cunningham S., Goodman-Bacon A., Sant'Anna P.H.C. (2025).

[29] Halliday K.E., Witek-Mcmanus S.S., Opondo C. (2020). Impact of school-based malaria case management on school attendance, health and education outcomes: a cluster randomised trial in southern Malawi. BMJ Glob Health.

[30] Cohee L.M., Opondo C., Clarke S.E. (2020). Preventive malaria treatment among school-aged children in Sub-Saharan Africa: a systematic review and meta-analyses. Lancet Global Health.

[31] Staedke S.G., Maiteki-Sebuguzi C., Rehman A.M. (2018). Assessment of community-level effects of intermittent preventive treatment for malaria in schoolchildren in Jinja, Uganda (START-IPT trial): a cluster-randomised trial. Lancet Global Health.

[32] Temperley M., Mueller D.H., Njagi J.K. (2008). Costs and cost-effectiveness of delivering intermittent preventive treatment through schools in western Kenya. Malar J.

[33] Habyarimana F., Ramroop S. (2020). Prevalence and risk factors associated with malaria among children aged six months to 14 years old in Rwanda: evidence from 2017 Rwanda malaria indicator survey. Int J Environ Res Public Health.

[34] World Bank (2018). National education profile: Tanzania 2018 update. https://www.epdc.org/sites/default/files/documents/EPDC_NEP_2018_Tanzania.pdf.

[35] (WHO) WHO. Crossing the Billion (2017). Preventive chemotherapy for neglected tropical diseases. https://www.who.int/neglected_diseases/resources/9789240696471/en/.

