# Supplementary material for: Implementation and effectiveness of intermittent preventive treatment in school aged children using dihydroartemisinin-piperaquine to reduce malaria burden: an implementation research of a cluster randomised trial in Tanzania
Source: eClinicalMedicine. 2025 Nov 7;90:103628. doi: 10.1016/j.eclinm.2025.103628 (PMC12639380; doi:10.1016/j.eclinm.2025.103628)
Supplement: Supplementary Figures and Tables [file mmc1.docx]

**Supplementary materials: Manuscript reference: eclinm-D-25-03314**

**Evaluation of the implementation and effectiveness of intermittent preventive treatment for malaria using dihydroartemisinin-piperaquine on reducing malaria burden in school aged children in Tanzania: an implementation research of a cluster randomised trial**

Geofrey Makenga,PhD^1,6*^, Bruno Mmbando, PhD^1^, Misago D. Seth, PhD^1^, Vito Baraka, PhD^1^, Daniel Chale, MSc,^1^, Filbert Fransis, PhD^1^, Athanas D. Mhina, MPhil^1^, Edwin Liheluka, MSc^1^, Daniel T.R. Minja, PhD^1^, Mercy Chiduo, PhD^1^, George Mtove, PhD^1^, Celine Mandara, PhD^1^, Samwel Gesase, PhD^1^, Method Segeja, MSc^1^, Mathias Kamugisha, MSc^1^, Paul M. Hayuma, MSc^1^, Joyce Mbwana, MSc^1^, Hillary Sebukoto, MD^1^, Anangisye Malabeja, MD^1^, Juma B. Tupa, BSc^1^, Sarah J. Ngede, CO^1^, Abdallah Lusasi, MPH^2^, Frank Chacky, MSc^2,6^, Anna David, MSc^2^, Sumaiyya G. Thawer, PhD^3,4^, Ally Mohamed, MPH^2^, Sijenunu Aaron, MSc ^2^, Samwel Lazaro, MPH^2^, Fabrizio Molteni, MMed^3,4^, Alex Nkayamba, MSc^5^, Hilde Bastiaens, PhD^6^, Jean-Pierre Van geertruyden, PhD^6#^, John P.A. Lusingu, PhD^1,7#^.

^1^National Institute for Medical Research, Tanga Centre, Tanga, Tanzania.

^2^National Malaria Control Program (NMCP), Tanzania.

^3^Swiss Tropical and Public Health Institute, Allschwill, Switzerland

^4^University of Basel, Basel, Switzerland

^5^Tanzania Medicine and Medical Devices Authority (TMDA)

^6^Global Health Institute, University of Antwerp, Antwerp, Belgium.

^7^Centre for Medical Parasitology, Institute of Medical Microbiology and Immunology, University of Copenhagen, Denmark

*Correspondence to Geofrey Makenga, Email: [geofmacky@gmail.com]

^#^ Share senior authorship

Contents

[**Supplementary materials** 1](#_Toc207990782)

[List of figures 2](#_Toc207990783)

[List of Tables 2](#_Toc207990784)

[**Safety of IPTsc** 3](#_Toc207990785)

[**Baseline characteristics.** 3](#_Toc207990786)

[**Impact of IPTsc on haemoglobin.** 5](#_Toc207990787)

[**Impact of IPTsc on clinical malaria** 7](#_Toc207990788)

[**Further information on individual schools involved on effectiveness monitoring and evaluation** 9](#_Toc207990789)

# List of figures

**F**igure S 1 ADR reports received per district council in an implementation study in 3 councils in Tanga Region, Tanzania 3

Figure S 2 Decline in malaria prevalence and increase in Haemoglobin levels per study arm among participants of an IPTsc implementation research in Tanzania 5

Figure S 3 Kaplan-Meier survival chart showing rate of developing malaria episodes among participants of an IPTsc implementation study in northesteran Tanzania 7

Figure S 4 Baseline malaria parasitaemia per school in schools involved on IPTsc study 9

Figure S 5 Round 1 IPTsc administration coverage per school and district 9

Figure S 6 Round 2 IPTsc administration coverage per school and district 10

Figure S 7 Round 3 IPTsc administration coverage per school and district 10

# List of Tables

Table S 1 Baseline characteristics of schoolchildren included in the effectiveness evaluation in an implementation study in 3 councils in Tanga Region, Tanzania 5

Table S3 Change in mean Haemoglobin from baseline at different follow up period among participants of an IPTsc implementation study in northeastern Tanzania. 7

Table S 4 Comparison of malaria prevalence reduction within intervention and control groups as a difference from baseline values with or without cluster adjustment among participants in an implementation study in north-eastern Tanzania 9

# **Safety of IPTsc**

Of Across all three IPTsc rounds, 165,748 DP doses were administered, with 49 adverse drug reaction (ADR) reports (0.03% of doses). These were distributed as follows: Handeni TC (26), Handeni DC (5), and Kilindi DC (18) (Supplementary Figure S1). Nearly half (49%, n=24) occurred in the first round. Most ADRs (87.8%, n=43) were non-serious. Of the six serious adverse events (SAEs), five involved headache, vomiting, and general weakness; all resolved following appropriate management. One SAE was a fatality due to suspected herbal intoxication, considered unrelated to IPTsc; the child had participated in all three rounds without prior side effects. All ADRs and SAEs were reviewed by the study supervision team in collaboration with the national regulatory authority’s pharmacovigilance unit.

**Figure S 1 ADR reports received per district council in an implementation study in 3 councils in Tanga Region, Tanzania**

**Baseline characteristics.**

A total of 3752 school children were enrolled for close monitoring for effectiveness evaluation (1971 in DP arm and 1781 in Control arm) (Table S1). The mean age was 10 years, with girls representing 51% of the population. About 65% of the participants reported a history of malaria in last month. Bed net ownership was 47%, while usage was at 27%.

The baseline malaria prevalence was 37% (n=1345). Fever prevalence was 11% (n=421), of whom 60% (n=252) had positive malaria test. Malaria prevalence varied with study arms and district councils. DP arm had 38.6% (n=748) and control had 34.1% (n=597) that differed to DP arm (p=0.005). Handeni DC had the highest malaria prevalence 58% (n=925) with all of its schools (clusters) classified as high strata (≥10% malaria prevalence), while Handeni TC was the lowest at 20% (n=98) which was more on DP arm (26%, n=72) than in control arm (12%, n=26). Though Kilindi DC had half of its schools classified as low strata, the overall malaria prevalence was 20% (n=322). The mean *Falciparum* parasite density was 6556.5/µL that was almost similar across study arms (Table 2), however, on considering asymptomatic cases only (i.e. with no fever) the mean *Pf* parasite density was 4683 p/uL. Low strata had almost half lower P.f parasite density (3234p/uL, n=29) compared to high strata areas (6632 p/uL, n=1264). The overall, anaemia prevalence was 36%(n=1363), where Handeni DC had highest anaemia prevalence at 53% (n=851) while Handeni TC had the lowest (though not far from Kilindi DC) anaemia prevalence at 22% (n=113) (Table ). Almost 70% of parents or guardians of the enrolled schoolchildren had primary education level, while the social economic parameters distributed almost equally to all categories (i.e. low 34%, median 33% and high 33%) (Table S1). Household (HH) characteristics included 58% with eaves open with a bit variation across study arms (54% in DP arm and 62% in the control arm). The median number of people in a HH was 7 (range:5-9), in which 3 (range:2-4) were school aged children and 1 (range:0-2) was under-five. The median number of sleeping places in a HH was 2 (range:1-2). These parameters were almost similar in both study arms (Table S1).

**Table S 1 Baseline characteristics of schoolchildren included in the effectiveness evaluation in an implementation study in 3 councils in Tanga Region, Tanzania**

| **Variable** | | **All** | **Study arms** | |
| --- | --- | --- | --- | --- |
|  |  |  | **DP** | **Control** |
| Number of children included | | 3752 | 1971 | 1781 |
| Mean age (SD) | | 10.3 (2.5) | 10.2 (2.5) | 10.3 (2.5) |
| Age group | 10-15 years, n (%) | 2305 (61.4) | 1218 (61.8) | 1087 (61.0) |
| Sex (Female), n(%) | | 1928 (51.4) | 1021 (51.8) | 907 (50.9) |
| History of malaria last month n (%) | | 2022 (64.8) | 1049 (67.8) | 973 (61.8) |
| Bednet ownership , n (%) | | 1481 (47.4) | 718 (46.4) | 763 (48.4) |
| Bednet usage, n (%) | | 847 (27.1) | 377 (24.3) | 470 (29.8) |
| Parental education |  |  |  |  |
|  | None, n (%) | 711 (22.7) | 337 (21.8) | 374 (23.7) |
|  | Primary, n (%) | 2167 (69.3) | 1071 (69.1) | 1096 (69.5) |
|  | Secondary and above, n (%) | 247 (7.9) | 141 (9.1) | 106 (6.7) |
| Social economic status | |  |  |  |
|  | low SES | 1291 (34.4) | 740 (37.5) | 551 (30.9) |
|  | Median SES | 1236 (32.9) | 632 (32.1) | 604 (33.9) |
|  | High SES | 1225 (32.6) | 599 (30.39) | 626 (35.2) |
| Household with open eaves n(%) | | 1820 (58.2) | 843 (54.4) | 977 (62.0) |
| Median (IQR) number of people in the HH | | 7 (5-9) | 7 (5-9) | 7 (6-9) |
| Median (IQR) number of children 5-15 years in the HH | | 3 (2-4) | 3 (2-4) | 3 (2-4) |
| Median (IQR) number of children under-five (yrs.) in the HH | | 1 (0-2) | 1 (0-2) | 1 (0-2) |
| Median (IQR) number of rooms in a HH | | 2 (2-3) | 2 ( 2-3) | 2 ( 2-3) |
| Median (IQR) number of sleeping places in a HH | | 2 (1-2) | 2 (1-2) | 2 (1-2) |
| Nutritional status |  |  |  |  |
| WAZ <-2zscore, n(%) | | 577 (15.4) | 285 (14.5) | 292 (16.4) |
| BMI <-2zscore, n(%) | | 198 (5.3) | 104 (5.3) | 94 (5.3) |
| **Study end points at baseline** |  |  |  |  |
| Prevalence of malaria, n (%) | All councils | 1345 (36.5) | 748 (38.6) | 597 (34.1) |
|  | Handeni DC | 925 (58.4) | 493 (61.9) | 432 (54.8) |
|  | Handeni TC | 98 (19.6) | 72 (26.2) | 26 (11.5) |
|  | Kilindi DC | 322 (20.1) | 183 (21.1) | 139 (18.9) |
| Fever n (%) | | 421 (11.2) | 263 (13.4) | 158 (8.9) |
| Malaria positive and Fever n(%) | | 252 (60.3) | 154 (58.8) | 98 (62.8) |
| Number of clusters in high strata* | All councils (n/N) | 18 /24 | 9/12 | 9/12 |
|  | Handeni DC (n/N) | 10/10 | 5/5 | 5/5 |
|  | Handeni TC (n/N) | 3/4 | 1/2 | 2/2 |
|  | Kilindi DC (n/N) | 5/10 | 3/5 | 2/5 |
| *P.f* Parasite density, mean (SD), All councils | | 6556.5 (19715.5) | 6887.2 (19501.0) | 6134.4 (19995.3) |
| *P.f* Parasite density no fever, all council, mean (SD), | | 4683.0 (17227.3) | 4274.3 (14474.6) | 5184.3 (20100.8) |
| *P.f* Parasite density, geometric mean (95% CI), All councils. | | 1341.0 (1210.5 - 1485.4) | 1350.0 (1173.7 - 1552.5) | 1329.7 (1144.3- 1545.1) |
| *P.m Parasite density,* mean (SD), *All councils (n=200)* | | 376.4 (669.2) | 330.2 (503.6) | 442.9 (852.2) |
| *P.o* Parasite density, mean (SD), all councils (n=24) | | 559.3 (859.9) | 568 (1005.9) | 542 (510.4) |
| Mean (SD) Haemoglobin | All councils | 12 .1 ( 1.7) | 12.0 (1.6) | 12.2 (1.8) |
| Prevalence of anaemia, n(%) | All councils | 1363 (36.3) | 736 (37.4) | 627 (35.2) |
|  | Handeni DC | 851 (52.8) | 406 (50.4) | 445 (55.1) |
|  | Handeni TC | 113 (22.1) | 85 (30.1) | 28 (12.2) |
|  | Kilindi DC | 399 (24.5) | 245 (27.8) | 154 (20.7) |
|  | |  |  |  |

*Legend*: SD- Standard deviation, IQR- Interquartile range, HH- Household, SES- Social economic status, WAZ- Weight for age z-score, BMI- Body Mass Index, DC- District council, *P.f- Plasmodium falciparum*

# **Impact of IPTsc on haemoglobin.**


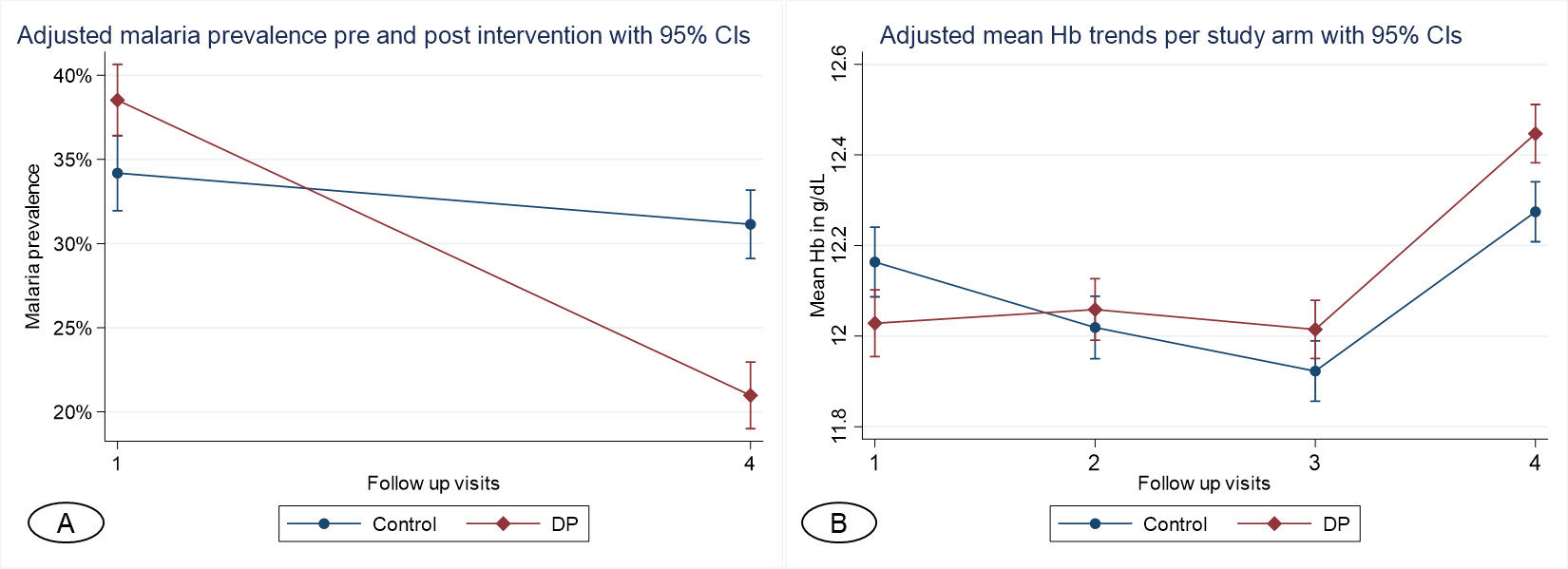


**Figure S 2 Decline in malaria prevalence and increase in Haemoglobin levels per study arm among participants of an IPTsc implementation research in Tanzania**

In the course of one year (month 12) of intervention implementation, in a linear mixed model, there were changes from baseline in haemoglobin levels (Hb) among participants in the intervention arm compared to those in control arm (0.31g/dL, 95%CI 0.2-0.4, p<0.001), this was on individual participant but fell short of significance on cluster adjustment (Table S3). Participants in high strata had a significant increase in haemoglobin at all scheduled visits, but this was not significant on cluster adjustment where only participants from low strata displayed an increase of 0.52 g/dl compared to Control at month 12. The trends on Hb increase across IPTsc rounds is as shown on Table S3 and Figure S2

**Table S3 Change in mean Haemoglobin from baseline at different follow up period among participants of an IPTsc implementation study in northeastern Tanzania.**

|  |  | **Study arms, Number tested (mean Hb g/dl)** | | | | | **mean difference of Hb (g/dl) change from baseline (DP vs Control)** | | | | | | | |
| --- | --- | --- | --- | --- | --- | --- | --- | --- | --- | --- | --- | --- | --- | --- |
| **Time point from baseline** |  | **DP** | | | **Control** | | **Adjusted* mean Hb change (95% CI)** | | **P-value** | **Cluster adjusted mean Hb change (95% CI)** | | **P-value** | | |
| **Intervention year** | Analysis | **N** | **mean (SD)** | | **N** | **mean (SD)** |  |  |  |  |  |  |  |  |
| Month 0 (baseline) | ITT | 1970 | 12.0 (1.6) | | 1781 | 12.2 (1.8) | NA | NA | NA | NA | NA | | NA |  |
|  | Modified ITT | 1647 | | 12.0 (1.6) | 1498 | 12.1 (1.8) | NA | NA | NA | NA | NA | | NA |  |
|  | High strata (ITT) | 1528 | | 11.8 (1.6) | 1341 | 11.9 (1.8) | NA | NA | NA | NA | NA | | NA |  |
|  | Low strata (ITT) | 442 | | 12.8 (1.6) | 440 | 13.1 (1.4) | NA | NA | NA | NA | NA | | NA |  |
|  |  |  | |  |  |  |  |  |  |  |  | |  |  |
| Month 4 | ITT | 1816 | | 12.1 (1.5) | 1783 | 12.0 (1.5) | 0.18 | (0.1-0.3) | **0.001** | 0.18 | (-0.3-0.6) | | 0.423 |  |
|  | Modified ITT | 1453 | | 12.1 (1.5) | 1370 | 12.1 (1.5) | 0.12 | (0.0-0.2) | **0.032** | 0.12 | (-0.1-0.5) | | 0.588 |  |
|  | High strata (ITT) | 1377 | | 11.9 (1.5) | 1352 | 11.7 (1.5) | 0.21 | (0.1-0.3) | **0.001** | 0.21 | (-0.3-0.8) | | 0.453 |  |
|  | Low strata (ITT) | 439 | | 12.7 (1.3) | 431 | 12.9 (1.1) | 0.03 | (-0.2-0.2) | 0.714 | 0.03 | (-0.5-0.5) | | 0.896 |  |
|  |  |  | |  |  |  |  |  |  |  |  | |  |  |
| Month 8 | ITT | 1858 | | 12.0 (1.4) | 1712 | 11.9 (1.5) | 0.23 | (0.1-0.3) | **<0.001** | 0.23 | (-0.1-0.5) | | 0.124 |  |
|  | Modified ITT | 1504 | | 12.0 (1.4) | 1361 | 11.9 (1.5) | 0.22 | (0.1-0.3) | **<0.001** | 0.22 | (-0.1-0.5) | | 0.141 |  |
|  | High strata (ITT) | 1427 | | 11.8 (1.4) | 1302 | 11.6 (1.4) | 0.30 | (0.2-0.4) | **<0.001** | 0.30 | (-0.1-0.7) | | 0.095 |  |
|  | Low strata (ITT) | 431 | | 12.6 (1.3) | 410 | 12.9 (1.2) | -0.05 | (-0.2-0.1) | 0.627 | -0.05 | (-0.5-0.4) | | 0.850 |  |
|  |  |  | |  |  |  |  |  |  |  |  | |  |  |
| Month 12 | ITT | 1818 | | 12.5 (1.4) | 1718 | 12.3 (1.5) | 0.31 | (0.2-0.4) | **<0.001** | 0.31 | (-0.1-0.7) | | 0.140 |  |
|  | Modified ITT | 1647 | | 12.4 (1.4) | 1498 | 12.3 (1.5) | 0.28 | (0.2-0.4) | **<0.001** | 0.28 | (-0.1-0.7) | | 0.184 |  |
|  | High strata (ITT) | 1384 | | 12.3 (1.4) | 1306 | 12.1 (1.5) | 0.23 | (0.1-0.4) | **<0.001** | 0.23 | (-0.3-0.7) | | 0.377 |  |
|  | Low strata (ITT) | 434 | | 13.1 (1.3) | 412 | 12.9 (1.3) | 0.52 | (0.3-0.7) | **<0.001** | 0.52 | (0.4-0.6) | | **<0.001** |  |
| *Legend: Adjusted* for individual child random-effects parameters, ITT= Intention to Treat, ICC= interclass correlation estimated following xtmixed model in ITT.* | | | | | | | | | | | | | | |

# **Impact of IPTsc on clinical malaria**

Survival analysis showed significant lower number of malaria episodes in the intervention compared to the control arm (log-rank, chi^2^=30.1, p<0.001) (Figure S2A). The high strata had stronger impact on survival analysis (log-rank, chi^2^=37.5,p<0.0001) (Figure S2B) compared to low strata that had no significant difference on number of malaria episodes between intervention and control arms (log-rank, chi^2^=0.11, p=0.7450) (Figure S2C).


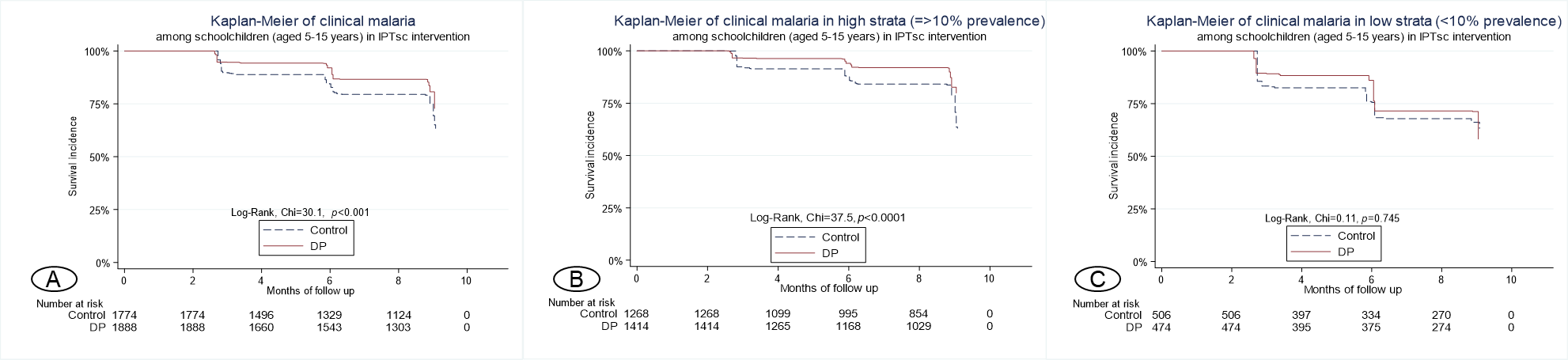


**Figure S 3 Kaplan-Meier survival chart showing rate of developing malaria episodes among participants of an IPTsc implementation study in northesteran Tanzania**

**Table S 4 Comparison of malaria prevalence reduction within intervention and control groups as a difference from baseline values with or without cluster adjustment among participants in an implementation study in north-eastern Tanzania**

| **A. Within group comparison on malaria prevalence change from baseline (Month0-Month12) adjusted by cluster (school), at intraclass corr of 0.2** | | | | | | | | | | | | | | | | | | | | | | | | | | |
| --- | --- | --- | --- | --- | --- | --- | --- | --- | --- | --- | --- | --- | --- | --- | --- | --- | --- | --- | --- | --- | --- | --- | --- | --- | --- | --- |
|  | | **DP** | | | | | | | | | | | **Control** | | | | | | | | | | | | | |
|  | | Baseline (M0) | | | Month 12 | | | Difference (M0-M12) | | | | | Baseline (M0) | | | | Month 12 | | | | Difference (M0-M12) | | | | | |
|  | | N | Prev% | | N | Prev % | | Diff % (95%CI) | | | p-value | | N | | Prev % | | N | Prev % | | | Diff % (95%CI) | | | p-value | | |
|  | |  |  | |  |  | |  |  | |  | |  | |  | |  |  | | |  |  | |  | | |
| ITT (unadjusted-crude) | | 1939 | 38.6 | | 1818 | 20.7 | | 17.8 | (15.0-20.7) | | **<0.001** | | 1749 | | 34.1 | | 1718 | 31.1 | | | 3 | (-0.0-6.2) | | 0.055 | | |
|  | |  |  | |  |  | |  |  | |  | |  | |  | |  |  | | |  |  | |  | | |
| ITT (k=12) | | 1939 | 38.6 | | 1818 | 20.7 | | 17.8 | (14.9-34.2) | | **0.036** | | 1749 | | 34.1 | | 1718 | 31.1 | | | 3.0 | (-14.2-20.3) | | 0.729 | | |
| Modified ITT(k=12) | | 1625 | 37.8 | | 1647 | 20.8 | | 17.0 | (0.5-33.3) | | **0.046** | | 1472 | | 33.9 | | 1498 | 30.8 | | | 3.0 | (-14.3-20.4) | | 0.730 | | |
| ITT-high strata(k=9) | | 1504 | 48.5 | | 1384 | 27.2 | | 21.3 | (1.4-41.2) | | **0.041** | | 1316 | | 44.1 | | 1306 | 40.3 | | | 3.9 | (-17.2-24.9) | | 0.719 | | |
| M-ITT-high strata (k=9) | | 1269 | 47.1 | | 1286 | 26.6 | | 20.5 | (0.6-40.4) | | **0.048** | | 1118 | | 43.6 | | 1141 | 40.0 | | | 3.6 | (-17.6-24.8) | | 0.740 | | |
| ITT-low strata (k=3) | | 435 | 4.4 | | 434 | 0.2 | | 4.1 | (-6.7-15.0) | | 0.458 | | 433 | | 3.7 | | 412 | 1.9 | | | 1.7 | (-10.3-13.8) | | 0.776 | | |
| M-ITT-low strata (k=3) | | 356 | 4.5 | | 361 | 0.3 | | 4.2 | (-6.8-15.2) | | 0.457 | | 354 | | 3.4 | | 357 | 1.7 | | | 1.7 | (-9.7-13.2) | | 0.770 | | |
|  | |  |  | |  |  | |  |  | |  | |  | |  | |  |  | | |  |  | |  | | |
| Avg cluster size, CV -ITT | | 161.6, 0.14 | | | 151.5, 0.09 | | |  |  | |  | | 145.7, 0.18 | | | | 143.2, 0.18 | | | |  |  | |  | | |
| Avg cluster size, CV- M-ITT | | 135.4, 0.13 | | | 137.2, 0.13 | | |  |  | |  | | 122.7, 0.21 | | | | 124.8, 0.21 | | | |  |  | |  | | |
| **B. Proportional difference of malaria prevalence change from baseline in DP arm compared to Control arm, determined on linear mixed model** | | | | | | | | | | | | | | | | | | | | | | | | | | |
|  | **Study arms, Number tested (prev %)** | | | | | | **Proportional difference of malaria prevalence change from baseline (DP vs Control)** | | | | | | | | | | | | | | | | | | **% Attributable reduction by IPTsc *** | |
|  |  |  |  |  |  |  | **Adjusted* prevalence change % (95% CI)** | | | **P-value** | | **Council adjusted prevalence change (95% CI)** | | | | **P-value** | | | **Cluster adjusted prevalence change (95% CI)** | | | | **P-value** | |  |  |
| Analysis M0-M12 | **DP** | | | **Control** | | |  |  |  |  |  |  |  |  |  |  |  |  |  |  |  |  |  |  |  |  |
|  |  | | |  | | |  |  | |  | |  | |  | |  | | |  |  | | |  | |  |  |
| ITT (k*=12) | 1818 (20.7) | | | 1718(31.1) | | | -14.5 | (-17.9--11.0) | | **<0.001** | | -14.5 | | (-25.2--3.8) | | **0.008** | | | -14.5 | (-22.0--6.9) | | | **<0.001** | | 81.5 |  |
| Modified ITT(k*=12) | 1647 (20.8) | | | 1498 (30.8) | | | -13.9 | (-17.5--10.3) | | **<0.001** | | -13.9 | | (-24.4--3.4) | | **0.009** | | | -13.9 | (-21.4--6.4) | | | **<0.001** | | 81.8 |  |
| ITT-high strata(k*=9) | 1457 (25.8) | | | 1319 (39.9) | | | -17.3 | (-21.7--12.9) | | **<0.001** | | -17.3 | | (-26.3--8.3) | | **<0.001** | | | -17.3 | (-25.8--8.8) | | | **<0.001** | | 81.2 |  |
| M-ITT-high strata (k*=9) | 1344 (25.4) | | | 1152 (39.6) | | | -17.0 | (-21.5--12.4) | | **<0.001** | | -17.0 | | (-25.6--8.3) | | **<0.001** | | | -17.0 | (-25.5--8.5) | | | **<0.001** | | 82.9 |  |
| ITT-low strata (k*=3) | 361 (0.3) | | | 399 (2.0) | | | -2.4 | (-5.4-0.6) | | 0.117 | | -2.4 | | (-4.4--0.4) | | **0.019** | | | -2.4 | (-5.8-1.0) | | | 0.175 | | 58.5 |  |
| M-ITT-low strata (k*=3) | 303 (0.3) | | | 346 (1.7) | | | -2.5 | (-5.7-0.7) | | 0.124 | | -2.5 | | (-4.1--0.9) | | **0.002** | | | -2.5 | (-6.3-1.3) | | | 0.199 | | 59.5 |  |
|  |  | | |  | | |  |  | |  | |  | |  | |  | | |  |  | | |  | |  |  |
| Model ICC % (95%CI)* | | | | | | | 37% | (34-40%) | |  | | 37% | | (30-44%) | |  | | | 37% | (31-43%) | | |  | |  |  |
| Model ICC-high strata % (95%CI)* | | | | | | | 30% | (26-34%) | |  | | 30% | | (23-37%) | |  | | | 30% | (25-36%) | | |  | |  |  |
| Model ICC -low strata% (95%CI)* | | | | | | | 0% |  | |  | | 0% | |  | |  | | | 0% |  | | |  | |  |  |
| *KEY:k*= number of clusters (schools) per study arm, Adjusted* for child random-effects parameters, ITT= Intention to Treat, ICC= interclass correlation estimated following xtmixed model in ITT* | | | | | | | | | | | | | | | | | | | | | | | | | | |

# **Further information on individual schools involved on effectiveness monitoring and evaluation**


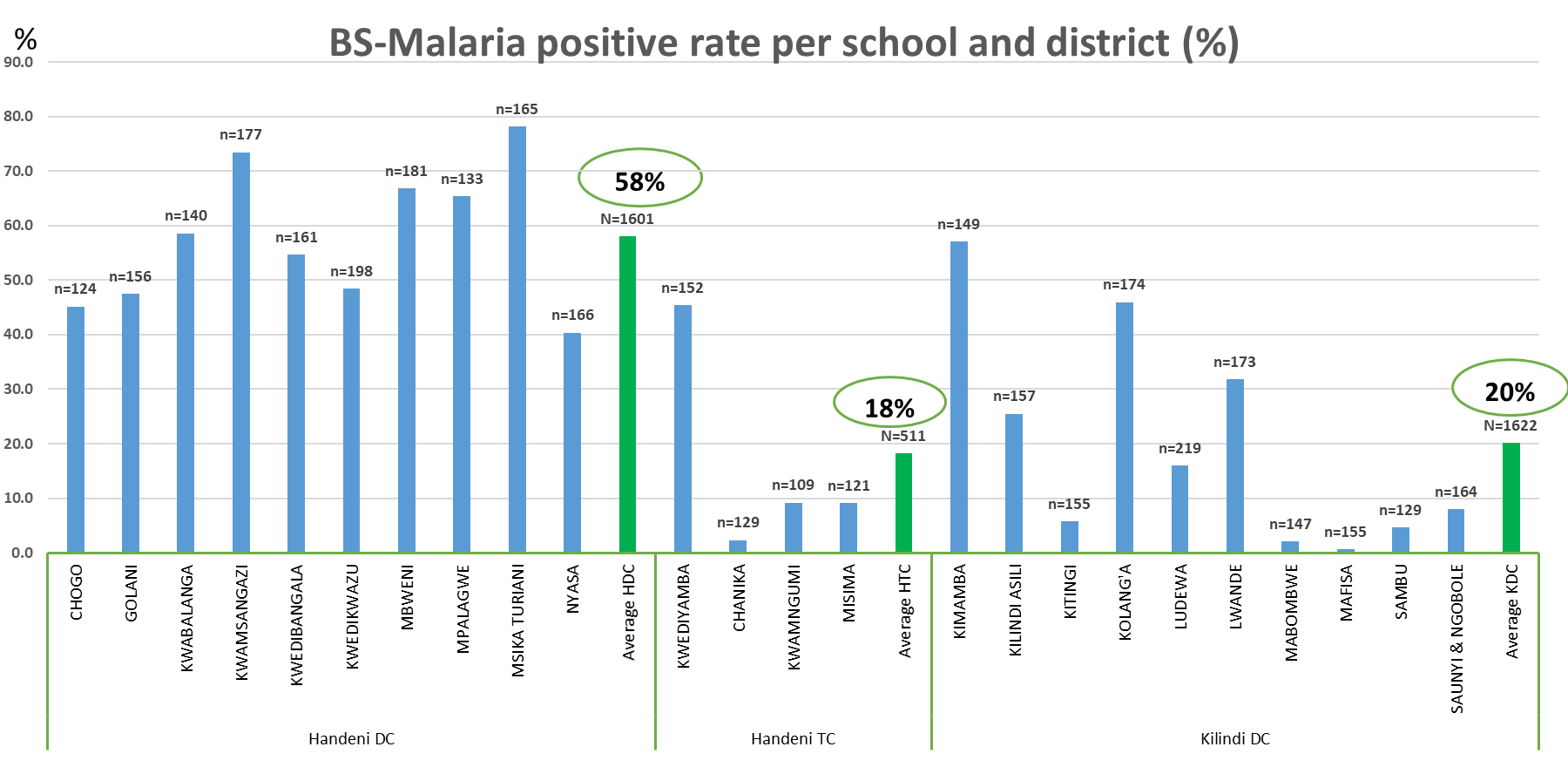


**Figure S 4 Baseline malaria parasitaemia per school in schools involved on IPTsc study**


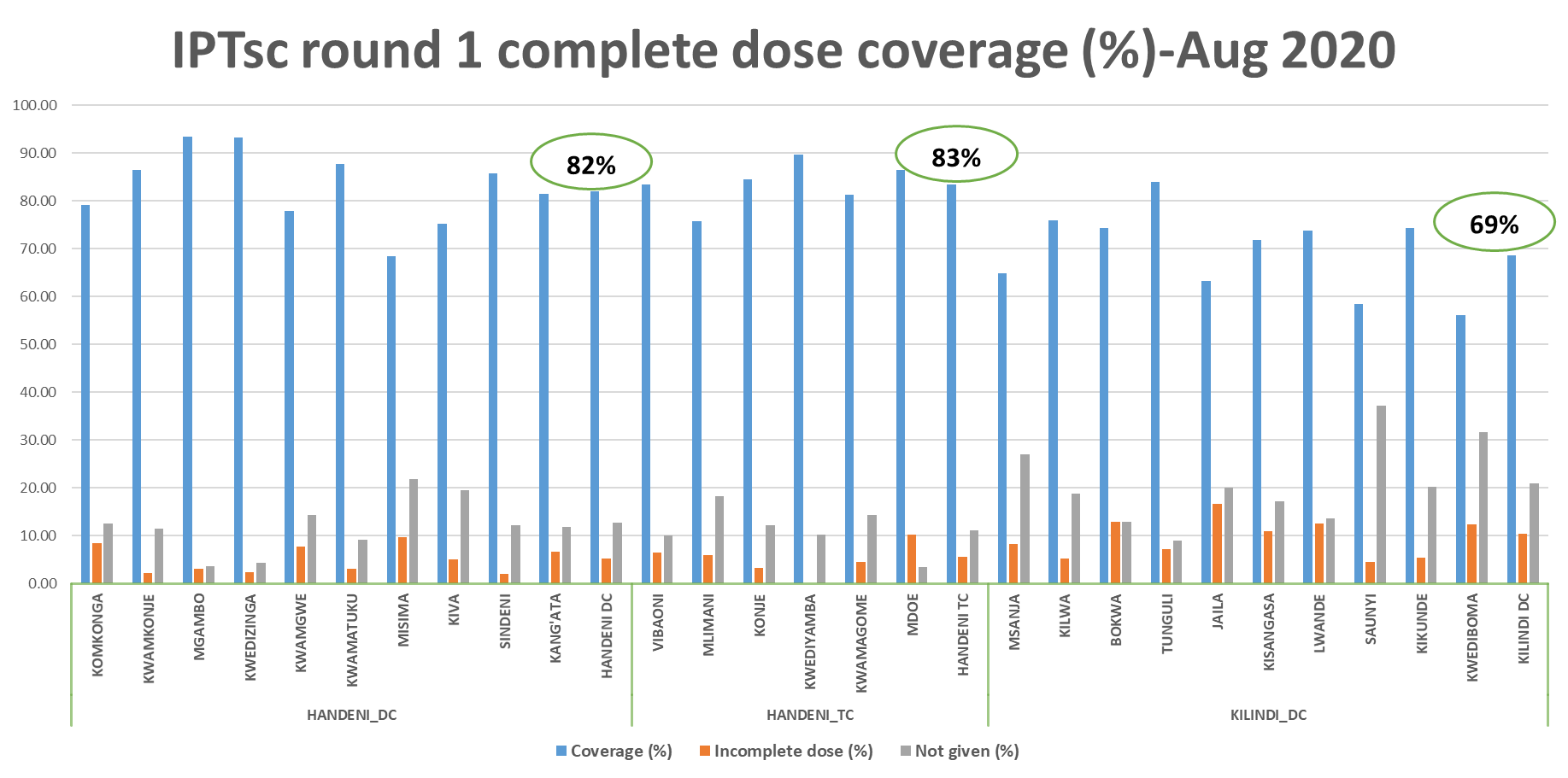


**Figure S 5 Round 1 IPTsc administration coverage per school and district**


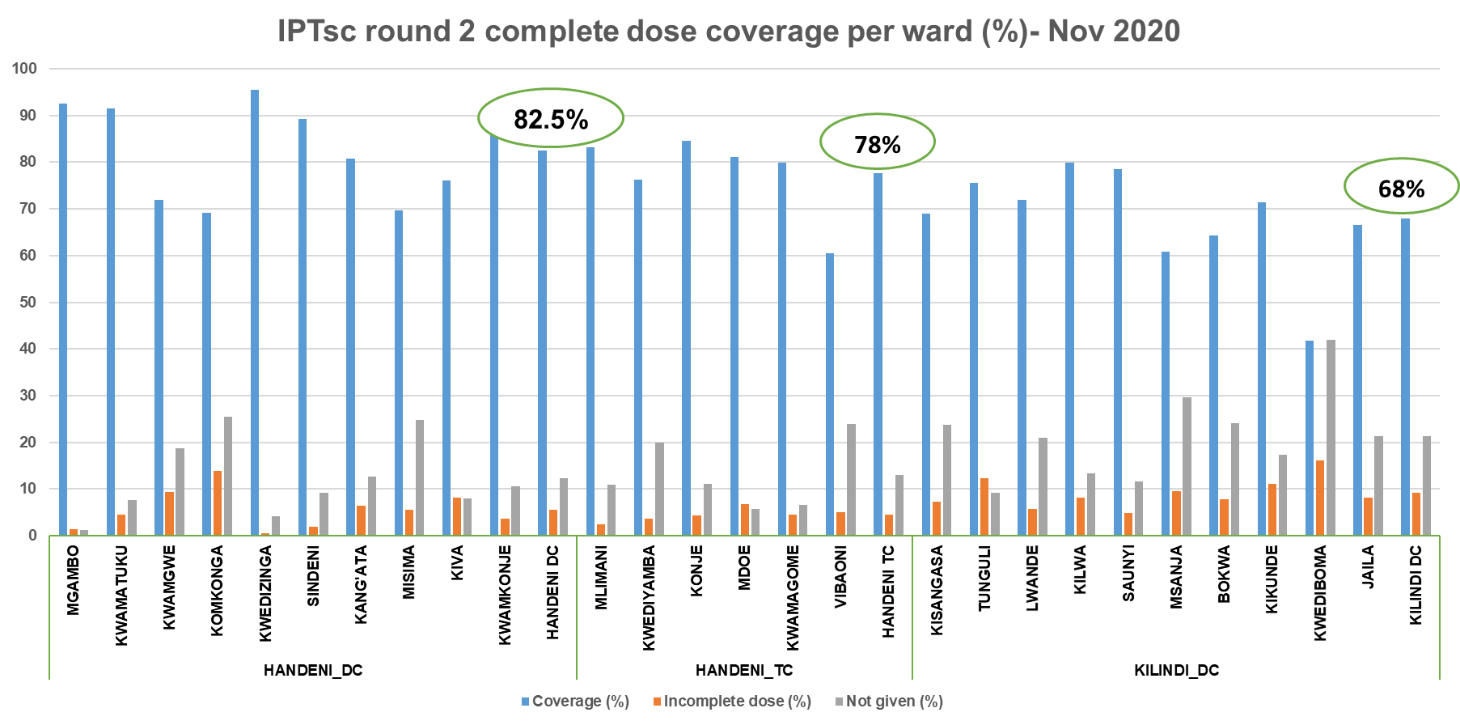


**Figure S 6 Round 2 IPTsc administration coverage per school and district**


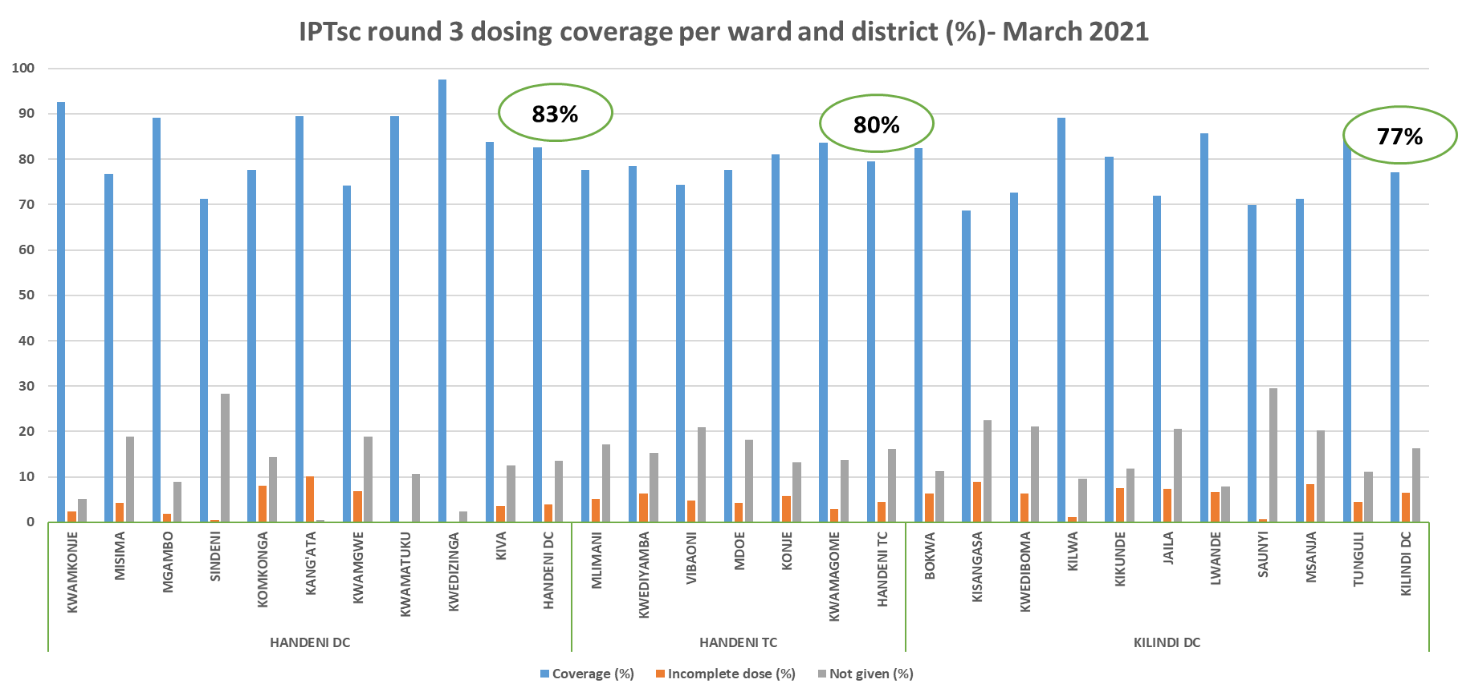


**Figure S 7 Round 3 IPTsc administration coverage per school and district**
